# An ecological analysis of snakes captured by C.J.P. Ionides in eastern Africa in the mid-1900s

**DOI:** 10.1038/s41598-020-61974-4

**Published:** 2020-03-20

**Authors:** Richard Shine, Stephen Spawls

**Affiliations:** 1 Department of Biological Sciences, Macquarie University, NSW 2109 Australia; 2 7 City College Norwich, Ipswich Road, Norwich, NR2 2LJ UK

**Keywords:** Behavioural ecology, Conservation biology, Forest ecology, Tropical ecology

## Abstract

Historical data can clarify ecological attributes of fauna in sites that have subsequently been altered by anthropogenic activities. We used the 1960s notebooks of pioneering East African naturalist C.J.P. Ionides to extract quantitative information on captures of 484 snakes of five species (rhinoceros vipers *Bitis nasicornis*, black mambas *Dendroaspis polylepis*, Jameson’s mambas *D. jamesoni*, water cobras *Naja annulata*, and eastern forest cobras *N. subfulva*). High capture rates suggest high abundances of all species. The relative numbers of each species collected changed over the years and differed seasonally, reflecting targeting by Ionides. Sex ratios and age-class distributions differed among species and were affected by factors such as month of collection and time of day. Habitat use was affected by species, sex and body size: for example, arboreality became less common with increasing body size in the rhinoceros viper and black mamba, and males were found in arboreal sites more often than were females. In both *D. jamesoni* and *D. polylepis*, adult males and females were recorded together in September-October, suggesting reproductive activity at this time of year. Although fragmentary, the data from Ionides’ notebooks provide a unique glimpse into ecological patterns of snakes within an African landscape half a century ago.

## Introduction

For snakes, as for many kinds of organisms, our knowledge of ecological traits is based primarily on studies of a small subset of species from a limited set of locations. In particular, taxa from areas within the tropics have been understudied relative to those from temperate-zone habitats^1^. Although ecological research on African snakes is beginning to fill that knowledge gap^2–8^, many species, from temperate-zone as well as tropical areas, remain virtually unstudied in the wild. Those taxa include some of the world’s largest and most spectacular snakes, such as mambas, cobras and vipers. Research has been discouraged by logistical problems associated with travel and security, as well as by the difficulties inherent in studying large venomous snakes.

The challenge of understanding ecological characteristics of tropical snakes is exacerbated by rapid degradation of habitats by the growing human population, with consequent shifts, and often, declines, in abundance of predators such as snakes^9^. Although that problem is worldwide, it is especially acute in some regions of the tropics, where land-clearing for agriculture has transformed the landscape^10–12^ and affected aspects such as soil quality and rates of erosion^12–15^. Such changes may advantage some species of snakes, but disadvantage others^4,16–18^. To understand the situation prior to this kind of habitat change, we must rely on historical records. Ideally, researchers can quantify temporal shifts in organismal abundances and distributions by careful replication of earlier surveys^19^. Even when replication is impractical, however, an analysis of historical data may clarify important aspects of ecosystems in earlier times.

**Figure 1:**
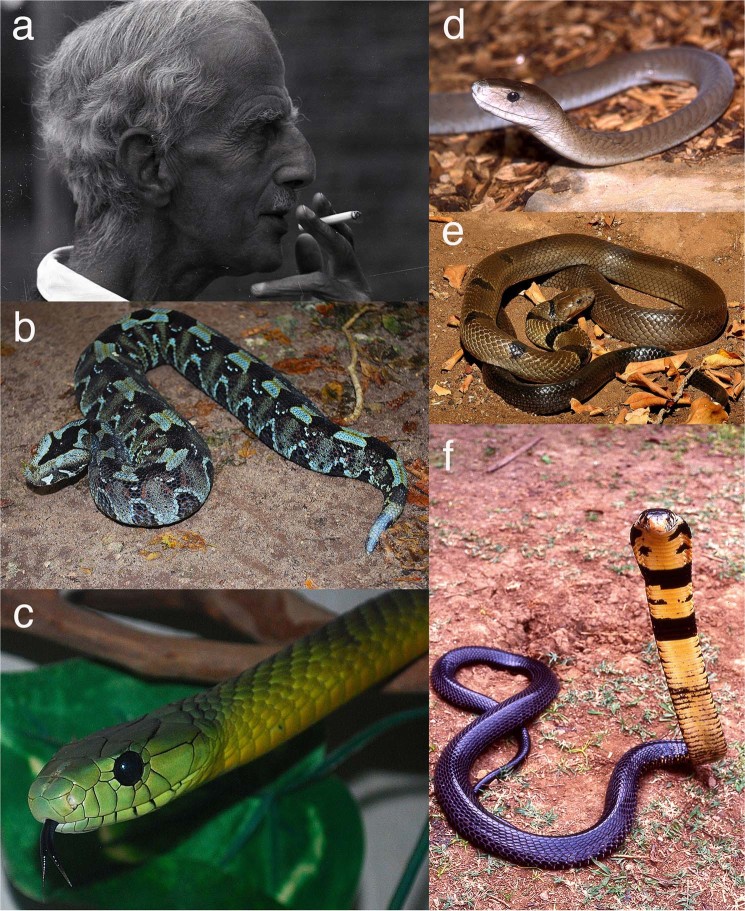
(**a**) C.J.P. Ionides, and the species of snakes for which data were available from his collection records: (**b**) rhinoceros viper, *Bitis nasicornis*; (**c**) Jameson’s mamba, *Dendroaspis jamesoni*; (**d**) black mamba, *Dendroaspis polylepis*; (**e**) water cobra, *Naja annulata*; (**f**) forest cobra, *Naja subfulva*. Photographs by S. Spawls (**a, b, c, d** and **f**), and W. Wuster (**e**), with permission.

In the present study, we report information on abundances, demography (age-class distributions, sex ratios) and habitat use in five species of large venomous snakes that were collected primarily in the 1950s and 1960s by one of the pioneers of African herpetology. Constantine John Philip Ionides (henceforth, “Ionides”, 1901 to 1968; see Fig. 1a) was a prominent figure in early scientific collections of herpetofauna in eastern Africa, and his colourful life has been the subject of two biographies^20,21^ and an autobiography^22^. During the latter part of his life, his focus shifted from big-game hunting to snake-collecting^23^. Although Ionides published few scientific papers^24^, he kept a detailed record of all of the large venomous snakes that he captured^25^. Those data are contained within a series of notebooks, some of which were available to us through the generosity of Jonathan Leakey. The pages relating to some of the species that Ionides collected have been removed from the notebooks, and cannot be located; they were presumably sent to C.R.S. Pitman to enable preparation of co-authored papers^26,27^. The remaining pages contain data on captures of six other east-African snake species. We have extracted information on five of those species (omitting *Elapsoidea sundevallii*, represented by only six records) from those notebooks.

The snakes for which we have collection data (location, date, time of day, size class, sex, habitat) represent only a small fraction of Ionides’ collections. A page in one of his notebooks includes a running lifetime tally of each species, totalling 10,964 venomous snakes overall. Of these, 6,633 were *Dendroaspis angusticeps*, 2,159 were *Bitis gabonica*, 386 were *Naja n. nigricollis*, 354 were *B. arietans*, 314 were *Causus defilippii*, 312 were *Echis pyramidum*, and 122 were *Vipera hindii* (= *Montatheris hindii*). Thus, the five species for which we have examined capture records, which are not included in the above list of “most commonly collected” taxa, constitute less than 5% of Ionides’ total collections (i.e., 484 of 10,964).

## Materials and Methods

### Collecting localities

The two species of snakes that Ionides collected in the largest numbers (*D. angusticeps* and *B. gabonica*) came from the Makonde Plateau, in the Newala District of Tanzania, within a 20-km radius of his home^26^. In contrast, the species for which we provide data in the current analysis primarily were taken elsewhere, during targeted trips, each of a few weeks in duration, to other localities in east Africa. His *Bitis nasicornis* were collected from the Ituri Forest, Democratic Republic of the Congo (10 animals) and the Kakamega Forest in Kenya (67 animals). The Kakamega Forest was the only site in which he collected *D. jamesoni*. In contrast, he caught *D. polylepis* from a variety of sites in Kenya, although the species was not found on the Makonde Plateau. *Naja subfulva* was captured from the Kakamega Forest in Kenya (*N* = 6), the Kisumu region of Kenya (*N* = 8), the Ituri Forest in the Belgian Congo (*N* = 4), Mpungulu in Northern Rhodesia (*N* = 1), and close to Ionides’ home in Newala, Tanzania (*N* = 9). Other Tanzanian specimens came from Liwale (*N* = 3) and Tukuya (*N* = 5). Lastly, *Naja annulata* was collected during trips to the southern end of Lake Tanganyika, in Tanzania (*N* = 8) and Zambia (*N* = 67).

### Species

All five species are large and highly venomous snakes; Ionides targeted them because he was able to sell live specimens to zoos, and to scientific institutions for their venom^26^. One species (*Bitis nasicornis*) is a heavy-bodied viperid, an ambush predator that inhabits forest and does not enter savanna; unlike all other members of the genus, it is partly arboreal. The other four are more slender-bodied elapids (see Fig. 1). *Dendroaspis polylepis* is the largest African elapid, reaching at least 3.2 m; it is a fast-moving diurnal snake, widespread in the savannas of Africa, confident in defence, with a bad but largely unjustified reputation. *Dendroaspis jamesoni* is also large, up to 2.64 m, but is a more secretive diurnal inhabitant of the forests of central Africa, from western Kenya west to Nigeria. *Naja annulata* is a poorly known elapid that seems to spend much of its time in water, both lakes and rivers, hunting fish by day. Those collected by Ionides were of an eastern subspecies, *Naja annulata stormsi*, largely confined to Lake Tanganyika, although the nominate subspecies extends west right through the forest to Cameroon. *Naja subfulva*, recently redefined, is a large alert diurnal cobra, reaching 2.7 m, and variable in colour. It has a large but patchy distribution in forest, woodland and wooded savanna, from eastern South Africa north to Ethiopia, and west to northern Cameroon. Two of the species collected by Ionides have been subject to recent changes in nomenclature. The water cobra was long known as *Boulengerina annulata*, but it has since been transferred to the genus *Naja*^28^. The widely-distributed forest cobra “*Naja melanoleuca*” has recently been shown to consist of five lineages; the one captured by Ionides is *N. subfulva*^29^.

### Methods

Ionides’ collection dates span the period from 1945 to 1968, with most (446 of 484, = 92%) falling between 1958 and 1966. One-quarter of all records (125) came from 1965. Ionides undertook trips to collect particular species only when he had a specific order from his clients^26^, but the seasonal timing of trips also depended on logistical issues. He travelled away from his home between July and October because those are the low rainfall months in southeast Tanzania; and that timing allowed him to collect snakes on the Makonde Plateau for scientific institutions during the rainy season (November through to April or May in southern Tanzania). As a result, the seasonal timing of Ionides’ captures says little about seasonal activity of the snakes. We nonetheless report these and related variables, because of their historical interest and to enable future comparisons with other datasets.

Ionides offered financial rewards to people from local villages to find snakes and alert him to their presence^26^. He then travelled to the site to collect the animal^25^. The time at which a snake was caught thus may say little about when the animal was active; hours may have elapsed between the time the animal was sighted and the time that Ionides captured it, especially since he was unable to walk long distances, and instead had to be transported in a specially-constructed wheelchair^25^. Another important bias in the notebook records was that Ionides collected mainly large snakes, and he did not record all of the juveniles that were reported to him^26^. Hence, his data overestimate the proportion of adult animals among the total sample that he had the opportunity to collect. The magnitude of this bias differed among species, however: he notes that few juvenile mambas were seen, whereas his samples of viperids contained animals of a wide range of body sizes^26^.

**Figure 2:**
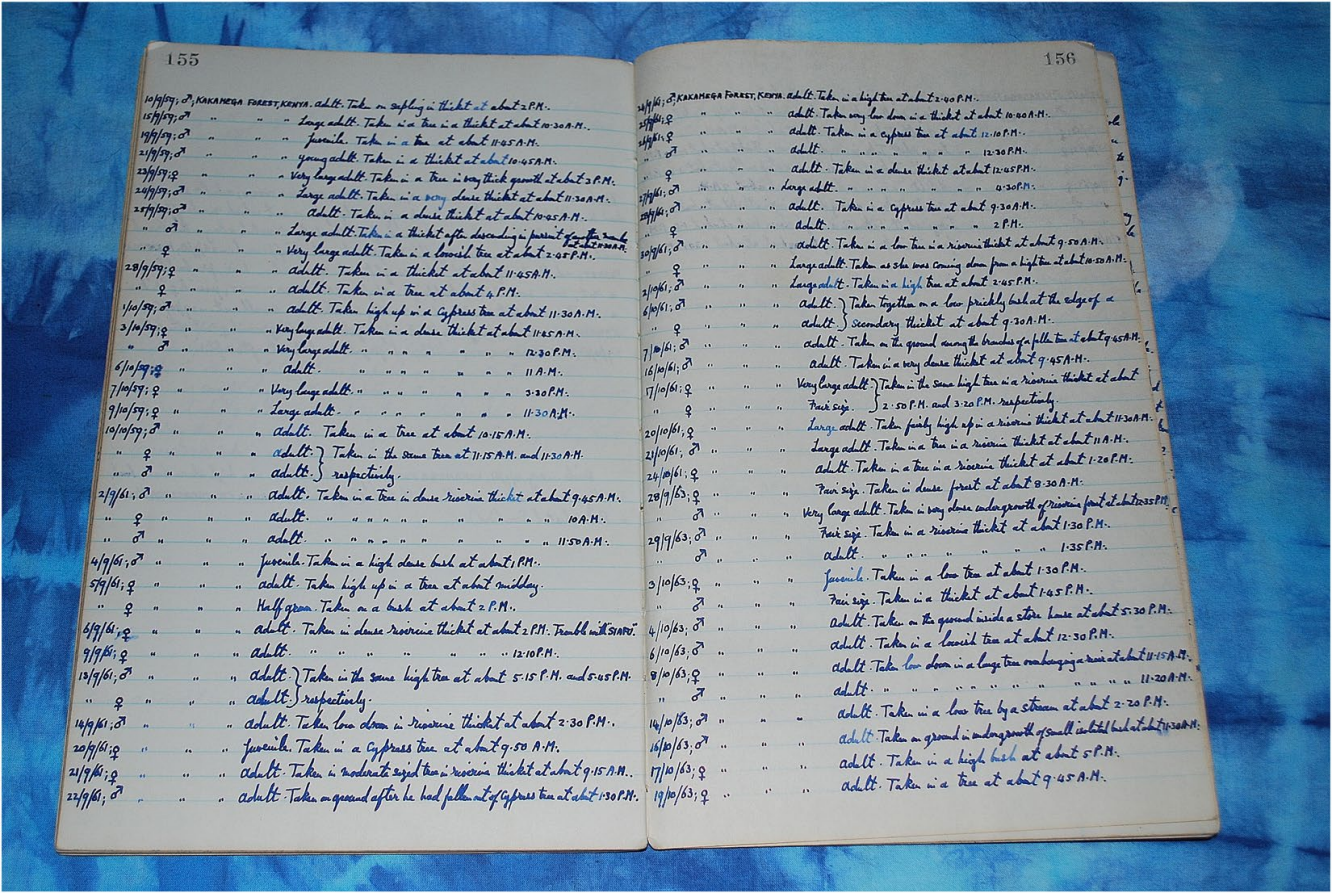
A page from one of Ionides’ notebooks, showing the nature of data that he recorded at capture events for one of the targeted species (*Dendroaspis jamesoni*).

Whenever Ionides captured a snake he recorded its locality (general region only), date and time of capture, sex and life stage^26^ (Fig. 2). His life-stage classification was as follows: “adult”, estimated hatched or born more than two years previously; “fair-size”, born or hatched two years previously; “half-grown”, born or hatched the previous year; and “juvenile”, born or hatched the same year. He also provided brief notes as to the habitat in which the snake was captured – for example, if it was in water or up a tree (and for *B. nasicornis* only, he recorded heights above ground), and he noted if two snakes were found in the same location at the same time.

### Statistical methods

We entered all of the data from Ionides’ notebooks into a spreadsheet, and we used JMP 13.0 (SAS Institute, Cary, NC) to conduct statistical analyses. All variables conformed to normality of distributions and variance homogeneity, so no transformations were needed prior to analysis. For continuous dependent variables, we used ANOVA to investigate significant associations among variables. For nominal and ordinal dependent variables, we used logistic regression to explore the statistical significance of departures from relevant null hypotheses. We treated “life stage” as an ordinal variable. In analyses with multiple independent variables, we included all interaction terms between factors, but deleted non-significant interactions (*p* > 0.05) and recalculated main effects. We did not correct for artifactually significant results due to multiple testing, because of the subjectivity inherent in defining groups of related tests^30^.

## Results

After excluding data for *Elapsoidea* because of the small sample size, we were left with data for 77 *Bitis nasicornis*, 68 *Dendroaspis jamesoni*, 228 *D. polylepis*, 36 *Naja subfulva* and 75 *N. annulata*.

### Rates of capture

The most striking feature of Ionides’ records is the high daily rates of capture. For example, he collected 47 *D. polylepis* within a two-week period (in October 1965), 34 *D. jamesoni* over a six-week period (September-October 1961), 25 *N. annulata* over a two-week period (August 1958), and 23 *B. nasicornis* within a month (October 1961).

### Changes in species captured over the years

The relative numbers of each species collected varied strongly through time (nominal logistic regression with year as factor and species as dependent variable; χ^2^ = 921.56, *df* = 76, *p* < 0.0001). All of the early records (from 1945 to 1953) concern a single taxon (*D. polylepis*). In contrast, *N. annulata* and *D. jamesoni* each were taken in only three years (respectively, 1956, 1958, 1960; and 1959, 1961, and 1963). *Bitis nasicornis* was collected in 1954, 1959, 1961 and 1963. *Naja subfulva* was taken at lower rates over a longer period (1954 to 1967; *N* = 0 to 9 snakes per year).

### Capture month

**Figure 3:**
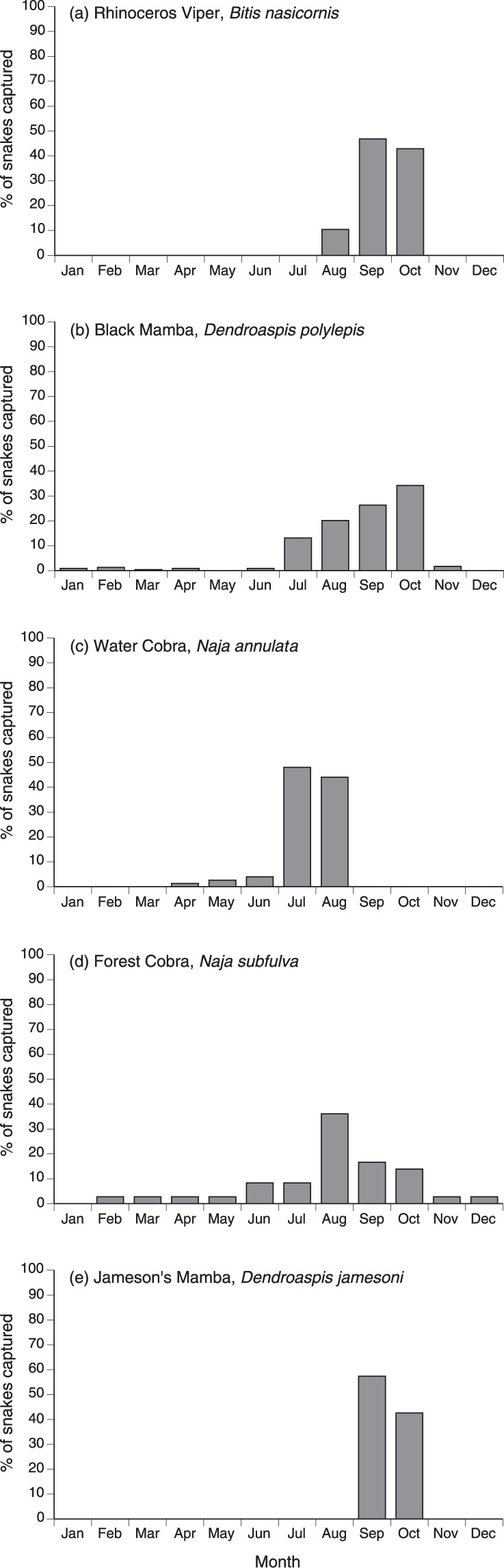
Monthly distribution of capture records for five species of snakes in east Africa, from the notebooks of C.J.P. Ionides.

The monthly distribution of captures differed among species (χ^2^ = 304.22, *df* = 33, *p* < 0.0001), but the primary capture period was August to October for all taxa (Fig. 3). All records of *B. nasicornis* and *D. jamesoni* fall within these three months, as do 81% of captures of *D. polylepis* and 67% of *N. subfulva*. In contrast, *N. annulata* were taken primarily in July (48%) and August (44%).

### Capture month vs. life stage

In a two-factor ANOVA with species and life stage as factors, the interaction between these two variables significantly affected month of collection (*F*_15,451_ = 1.82, *p* < 0.03). However, no relationship between life stage and month of capture was statistically significant when analyses were conducted separately for each species.

### Capture month vs. sex

Sex ratios of captured snakes did not differ significantly among months in the overall sample, but monthly variation was significant within *N. subfulva* (*F*_10,35_ = 19.50, *p* < 0.035). However, the divergence between sexes was minor (female-bias in September, male-bias in October) and unlikely to be biologically significant.

### Capture time of day

Except for 10 snakes that Ionides recorded as being captured in the evening (without specific times), all snakes were taken between 0730 h and 1900 h. There were no strong peaks or troughs within that period, but capture times differed among species. The relative numbers of captures in the morning (versus the afternoon) was 56% for *D. jamesoni*, 46.5% for *D. polylepis*, 34.2% for *B. nasicornis*, 30.6% for *N. subfulva*, and 20% for *N. annulata*. Those interspecific divergences are statistically significant (χ^2^ = 27.03, *df* = 4, *p* < 0.0001).

### Capture time of day vs. life stage

**Figure 4:**
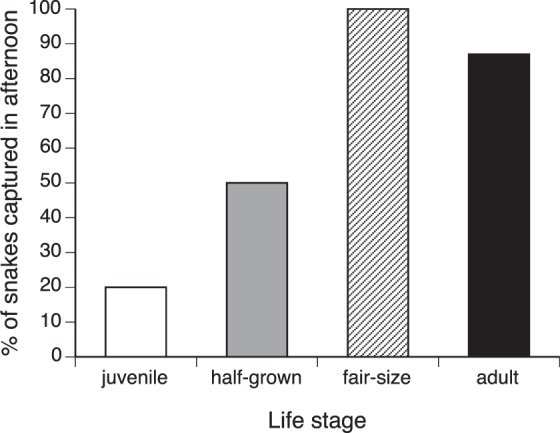
Times of day at which C.J.P. Ionides captured different age classes of snakes of five species. Data are combined for five species, because statistical analysis revealed no significant interspecific differences in these patterns (see text). The histograms show the proportion of total captures for each age class that were caught in the afternoon rather than the morning. Most captures of juvenile snakes were in the morning, whereas larger snakes were generally captured in the afternoon.

In a nominal logistic regression, capture time (AM vs. PM) was affected both by species (χ^2^ = 29.79, *df* = 4, *p* < 0.0001) and by life stage (χ^2^ = 7.11, *df* = 1, *p* < 0.008; interaction term NS so deleted). Figure 4 depicts the effect of life stage on time of capture, with a higher proportion of juveniles captured in the morning and older age classes captured in the afternoon.

### Species differences in relative numbers of each life stage

Ionides’ samples of all species were dominated by adults (from 61% in *B. nasicornis* to 87% in *D. jamesoni*), but the proportion of juvenile animals was highest in *N. subfulva* (19%) and lowest in *D. polylepis* (4%; treating life stage as an ordinal variable, species effect *F*_4,477_ = 3.62, *p* < 0.007).

### Sex ratio

Sex ratios of the collected snakes varied among species (logistic regression – χ^2^ = 16.12, *df* = 4, *p* < 0.003), being female-biased in *B. nasicornis* (59.7%) and *N. annulata* (62.7%) but male-biased in the terrestrial elapids (*D. polylepis* 40.8% female, *D. jamesoni* 47.1%, *N. subfulva* 41.7%).

**Figure 5:**
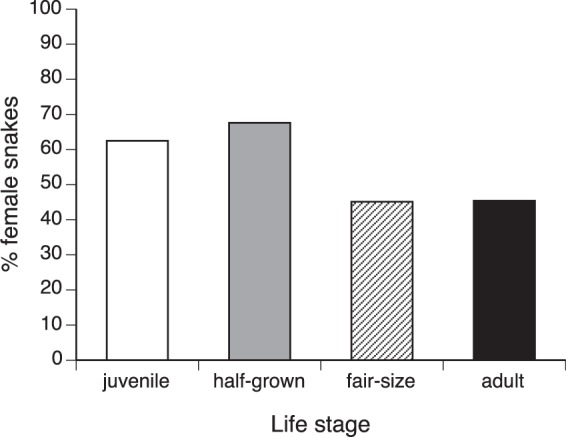
Ontogenetic (age-class) variation in sex ratios of snakes captured by C.J.P. Ionides. Data are combined for five species, because statistical analysis revealed no significant interspecific differences in these patterns (see text). The proportion of snakes that were females declined among larger size classes.

If we include life stage as well as species as factors in a nominal logistic regression, sex ratios are influenced by both of these variables (species effect χ^2^ = 16.09, *df* = 4, *p* < 0.003; life stage effect χ^2^ = 6.60, *df* = 2, *p* < 0.015; interaction NS so deleted; see Fig. 5). Samples of younger snakes were female-biased (62.5% females in juveniles, 67.6% in half-grown animals) whereas most larger snakes were males (45.0% female in fair-sized animals, 45.4% in adults). The trend was also statistically significant within *N. subfulva* (χ^2^ = 6.48, *df* = 3, *p* < 0.011).

### Habitat use

**Figure 6:**
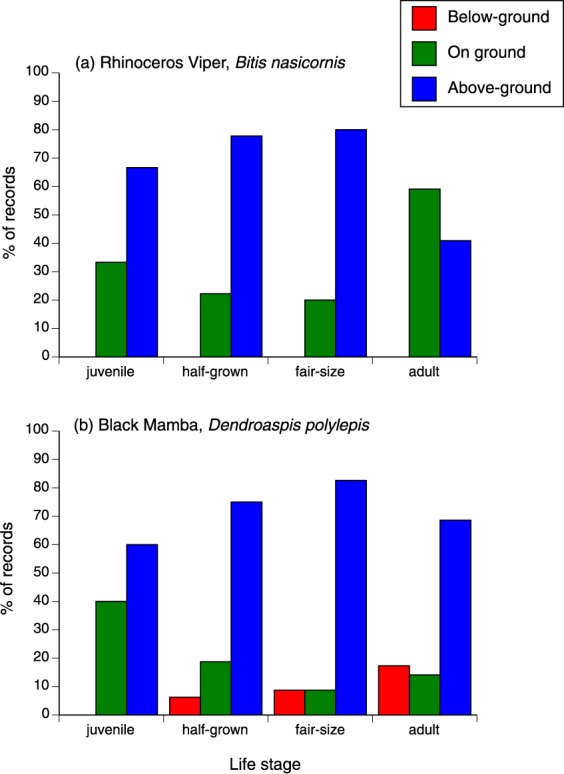
Ontogenetic (age-class) variation in habitat use of rhinoceros vipers *Bitis nasicornis*, and black mambas *Dendroaspis polylepis*, captured by C.J.P. Ionides. Arboreality was less common in larger rhinoceros vipers, and fossoriality was more common in larger black mambas.

For analysis, we divided habitats into four categories (arboreal, terrestrial, below-ground, in water). The species differed in terms of relative numbers within each of these categories (χ^2^ = 450.70, *df* = 12, *p* < 0.0001; interaction between species and life stage χ^2^ = 20.87, *df* = 12, *p* = 0.052). The species effect reflects a pattern in which the two mamba species were found primarily in trees, the water cobra was found in the water, the rhinoceros viper was found both on the ground and in trees, and the eastern forest cobra was found in all habitats. Looking separately within each species, the ontogenetic shift in habitat use was statistically significant within *B. nasicornis* (shift from arboreal to terrestrial sites with increasing age; χ^2^ = 8.73, *df* = 2, *p* < 0.015) and *D. polylepis* (adult snakes more often found in fossorial rather than arboreal sites; χ^2^ = 7.13, *df* = 2, *p* < 0.03; see Fig. 6).

A snake’s sex also affected its habitat choice (nominal logistic regression; species effect χ^2^ = 439.19, *df* = 12, *p* < 0.0001; sex effect χ^2^ = 8.88, *df* = 3, *p* < 0.035; interaction species*sex NS). The sex difference involved males being found in trees more often than were females (65.4 vs. 51.5%) and also, being found less often on the ground (21.9 vs. 12.5%).

For one species (*B. nasicornis*), Ionides also recorded height above the ground. No significant differences in mean height above ground were evident as a function of a snake’s sex (*F*_1,21_ = 0.79, *p* = 0.38) or its life stage (*F*_1,21_ = 2.64, *p* = 0.12; and interaction sex*life stage = NS).

### Co-occurrence of snakes

Records of snakes found together in the same site may reflect social interactions such as courtship, mating and male-male combat. For *D. polylepis*, Ionides recorded cases involving two adult males collected from the same antbear hole (October 1965), close to each other within a thicket (October 1959), and smoked out of the same tree (7 days apart, September 1967). A female was captured six days after a male was caught in the same tree (September 1965), a juvenile female and an adult male were both taken from under bark of the same dead tree (October 1965), and an adult male and female were taken from the same antbear hole (October 1965). For *Dendroaspis jamesoni*, Ionides recorded an adult male plus an adult female in the same trees on four occasions (October 1959, September 1961, October 1961, October 1963), and one additional case of two females (one adult, one fair-sized) in the same tree (October 1963). Lastly, two adult male *Naja subfulva* were dug out of the same termite mound two days apart in October 1963.

## Discussion

Ionides’ methods were designed to maximise the number of specimens that he could capture, not to provide unbiased samples of the abundance and distribution of snakes in his collection sites. He relied upon incidental sightings by others, captured animals only by day, and only targeted particular species when he had orders to fill, and at places where he could be assured of high rates of encounter. Thus, Ionides’ notebooks provide only snapshots of the ecology of snakes in east Africa in the 1950s and 1960s, but the data nonetheless can be used to clarify two main issues: the abundance of snakes, and non-random patterns in habitat use with respect to snake species, sex and life stage.

It is clear both from the present dataset, and from published summaries of Ionides’ collections of other snake species, that he was able to find and catch large venomous snakes at a rate that seems remarkable in light of other data. For example, Penner *et al*.^31^ noted that most field studies of *Bitis gabonica* have been based on total sample sizes of two to nine specimens, although those numbers increased markedly when snakes were also obtained from bushmeat markets or over long periods. In their own field study, Penner *et al*.^31^ found rhinoceros vipers (*B. rhinoceros*) by following troops of monkeys through the forest and listening for alarm calls. The researchers averaged 0.01 snake per hour and located a total of 41 individuals within a 770-ha study area (1 snake per 18.8 ha). During concurrent surveys for leaf-litter anurans, investigators found an average of 0.002 vipers per hour^31^. Luiselli^32^ estimated a mean population density of 0.10 individuals per hectare (range 0.01 to 0.30) for *B. nasicornis*, and Bombi *et al*.^33^ defined “high abundance” sites for *B. nasicornis* as those in which they found at least two individuals for every 10 hours in the field. For *Dendroaspis jamesoni*, intensive research resulted in capture of seven adult snakes over a 109-day period, and estimated a density of 0.11 adults per hectare^4^. Population densities at Ionides’ main collecting sites appear to have been higher. For example, he captured 973 green mambas (*D. angusticeps*) between March 1954 and January 1961within a radius of about 10 miles of his home in Newala^26^. The same paper reports catching 302 gaboon vipers (*B. gabonica*) in an area of 3 × 2 miles, and catching 217 specimens in 47 days (May to June 1961). Perhaps Ionides’ most remarkable record is of capturing 18 green mambas (*D. angusticeps*) and one boomslang (*Dispholidus typus*) in a three-and-a-half-hour period, and stopping only because his hand became tired^34^. The analyses in the present paper are based on smaller numbers, but the daily rates of capture nonetheless were high. Lacking data on current population densities of snakes in these collecting sites, or any way to translate capture rates to underlying population densities, all we can conclude is that large venomous snakes were abundant half a century ago. That may no longer be the case, at least for some taxa. For example, counts of *B. nasicornis* at a Nigerian study site fell by >90% over the period from 1995 to 2008, whereas counts of *D. jamesoni* remained stable over the same timeframe^9^.

The size-class distributions of Ionides’ snakes differ among species, likely reflecting biological factors (notably, differences in the ease of observing small specimens in different habitats) as well as his focus on larger specimens because of their commercial value. With respect to the two species that he captured in huge numbers near Newala, he notes that the greater abundance of juveniles in gaboon vipers versus green mambas was due to differences in encounter rates rather than due to his own size-selection. The data we analysed show similar patterns; that is, more juveniles in vipers than in mambas. Similar consistency between Ionides’ published comments on other species and our own analyses is evident in sex ratios – we found a female bias in rhinoceros vipers as in gaboon vipers, but a male bias in black mambas and Jameson’s mambas as in green mambas^26^. Studies on other elapid species have reported that females are encountered more frequently than males in viviparous species where females facilitate embryonic development by basking for long periods in exposed situations, whereas males are encountered more frequently in species in which males travel about extensively during mate-searching activities^35^. Such consistencies suggest that non-random sex ratios in Ionides’ collections reflect aspects of snake ecology rather than sampling errors. In subsequent work, Luiselli *et al*.^4^ and Luiselli and Akani^36^ have reported relatively equal numbers of males and females in field-collected samples of both *B. nasicornis* and *D. jamesoni*, but the two species differ in sexual size dimorphism: males grow larger in the mambas whereas females grow larger in the vipers. Importantly, observability of snakes is affected by feeding, as recently-fed animals selected more exposed sites, so were more obvious, and feeding frequencies differ between the sexes (e.g., gravid female *B. nasicornis* cease feeding^32^). These kinds of behavioural differences between the sexes plausibly explain non-random patterns in the sex ratios of animals collected by Ionides.

Ionides’ notebooks also provide rich detail about the circumstances of capture of 484 snakes. Strong biases in the timing of his collecting activities mean that we can say little about seasonality of activity or reproduction, but such biases should not affect data on issues such as non-random habitat use with respect to snake species, sex and body size. Broadly, the patterns that we detected are consistent with published results on other species of snakes in other parts of the world. For example, a relative scarcity of juvenile specimens in the relatively arboreal mambas may well reflect observability in this complex habitat, as noted by Ionides and Pitman^26^ for the green mamba *D. angusticeps*. These authors noted that mambas frequently bask in lower and more open areas of the trees in the morning, retreating to thicker foliage later in the day. Presumably smaller snakes would heat up more rapidly^37^, and hence retreat from open sites sooner; and would be more difficult to see within a dense tangle of vegetation. In contrast, juvenile rhinoceros vipers (*B. nasicornis*) are likely to remain sedentary, and observability of a snake in the ground, especially if encountered during agricultural activities, when the ground is being tilled, may be less dependent on the animal’s body size than would be the case for a snake in a tree.

Likewise, a reptile’s sex and body size often may affect its use of arboreal habitats^38,39^. The biomechanics of climbing favour elongate morphology and limit the maximum body size that can be supported by a slender branch^40^. Those functional constraints generate simple predictions: that arboreality should be more common (a) in more elongate species, (b) in smaller snakes within a species, and (c) in males not reproductive females, because gravid females are heavily distended with the clutch or litter, especially in viviparous species^41^. In keeping with those predictions, our analysis of Ionides’ data recorded arboreality more frequently (a) in slender-bodied species; (b) in smaller age classes within a species, and (c) in males rather than females. We note, however, that other factors might also drive non-random associations between a snake’s phenotype and its habitat use. For example, larger snakes may be less arboreal because they are less vulnerable to terrestrial predators, or because they have different thermoregulatory priorities, or because they target different prey types^2,4,38,42,43^.

In summary, some of the associations between ecological variables (time of day, habitat) and snake species, size, and sex likely reflect functional links that are mediated through interspecific and intraspecific variation in behavioural traits. Other non-random patterns within the dataset reflect the behaviour of Ionides (e.g., where and when he collected snakes) rather than of the snakes themselves. Teasing apart the proximate mechanisms involved, and documenting wider aspects of the ecology of these iconic snakes, remains a challenge for future researchers who follow in the footsteps (or rather, wheelchair tracks) of this intrepid pioneering snake biologist.

## Supplementary information

Supplementary information.

Supplementary information2.

## Data Availability

The datasets generated and analysed in this study are available from the corresponding authors on reasonable request.
