# Supplementary material for: An ecological analysis of snakes captured by C.J.P. Ionides in eastern Africa in the mid-1900s
Source: Sci Rep. 2020 Mar 20;10:5096. doi: 10.1038/s41598-020-61974-4 (PMC7083826; doi:10.1038/s41598-020-61974-4)
Supplement: Supplementary file 1 — Supplementary information. [file 41598_2020_61974_MOESM1_ESM.pdf]

right eye. Washed it with milk.
...ms": No treatment.
...atment.
...ent.
...l it with weak permanganate.
...ent.
...t.

...ment.
...t.
...hed it with water.
Serum injected.

Poisonous Front-Fanged Snakes from 1/1/45.

	31/12/62.	31/12/63.	31/12/64.	31/12/65.	31/12/66.	31/12/67.
B. FASCIATUS	—	—	—	—	1.	1.
E.S. GÜNTHERI	6.	6.	6.	6.	6.	6.
E.S. DECOSTERI	22.	22.	22.	22.	22.	22.
B.A. STORMSI	75.	75.	75.	75.	75.	75.
N.H. HAJE	3.	7.	11.	11.	11.	11.
N. MELANOLEUCA	19.	22.	31.	32.	34.	36.
N.N. PALLIDA	2.	2.	2.	2.	2.	2.
N.N. NIGRICOLLIS	56.	97.	139.	195.	259.	386.
P. GOLDII	1.	1.	1.	1.	1.	1.
D.J. KAIMOSAE	54.	77.	77.	77.	77.	77.
D. ANGUSTICEPS	1277.	1847.	2786.	3366.	6005.	6633.
O. HANNAH	—	—	—	—	16.	16.
N.N. KAOUTHIA	—	—	—	—	5.	5.
D.P. POLYLEPIS	20.	21.	30.	154.	154.	201.
P. PLATURUS	1.	1.	1.	1.	1.	1.
A.M. MICROLEPIDOTA	2.	2.	2.	2.	2.	2.
A. CORPULENTA	1.	1.	1.	1.	1.	1.
A.B. ROSTRATA	45.	48.	49.	49.	50.	50.
A.I. BIPOSTOCULARIS	—	—	2.	2.	2.	2.
V. R. SIAMENSIS	—	—	—	—	1.	1.
C. RHOMBEATUS	35.	35.	36.	36.	37.	37.
C. RESIMUS	1.	1.	2.	2.	2.	2.
C. DEFILIPPII	221.	233.	265.	277.	288.	314.
C. LICHTENSTEINII	1.	1.	1.	1.	1.	1.
V. HINDII	74.	74.	118.	122.	122.	122.
C. CERASTES	—	—	—	10.	10.	10.
B. WORTHINGTONI	7.	7.	7.	7.	7.	7.
B.A. ARIETANS	203.	246.	290.	314.	330.	354.
B.G. GABONICA	1057.	1260.	1553.	1757.	1974.	2159.
B. NASICORNIS	62.	77.	77.	77.	77.	77.
E.C. PYRAMIDUM	312.	312.	312.	312.	312.	312.
A. SQUAMIGER	21.	29.	29.	29.	29.	29.
A.N. NITSCHEI	1.	1.	1.	1.	1.	1.
E. COLORATUS	—	—	—	2.	2.	2.
A. RHODOSTOMA	—	—	—	—	8.	8.
TOTAL	3579.	4505.	5926.	6943.	9925.	10964.

Local names.— NDEMERA; NDEMALUNYOYI; MKOROWONJO; NAKEHUNGU; GOBOKO; RUWERO; ALASURHE; MAEN DON; KIPLOGOYON.

DENDROASPIS POLYLEPIS POLYLEPIS GÜNTHER, 1864.— Southern Brown-Mamba, Southern Black-Mamba.

Description. Head small, narrow, elongate, deep (i.e. not flat), straight-sided and moderately distinct from the neck; eye moderate, brown, with round pupil; body relatively slender, though it may be fairly thick in the middle in large specimens, and tapering towards head & tail. S.R. 21-25 (usually 23); V. 142-182; S.C. (paired) 105-127; anal divided; fangs very far forward in upper jaw with no other teeth near them; loreal absent; scales smooth & oblique, having a delicate bloom on them; 2 or 3 preoculars and an anterior subocular below them, which may occasionally be fused with the 3rd upper labial; 2 or 3 postoculars and a posterior subocular below; several upper temporals are in contact with the outer border of the parietal, 2nd upper labial large & in contact with prefrontal. (D.P. ANTINORII differs in having 2nd upper labial small & not in contact with prefrontal, and in average number of scale rows being 25 instead of 23).

Colour of adults, above, dark brown; olive usually darker on the back than on the sides; slate-coloured; lightish brown, sometimes faintly barred with darker brown; lead-grey. Underparts white. Colour of juveniles dull greyish green above, lighter below. Buccal membranes inside the mouth are black to bluish black.

Length of over 14' has been recorded in South Africa, but 7'6" to 9' is normal in East African adults.—7/3/54; MIHURU, MTWARA; ♀ over 10' taken.—5/4/61; VOI, KENYA; ♂ about 10'6" (3200 M.H.) taken.—9/11/62; KIMANA, K.C.; ♂; length 8'-10½"; tail 1'-9"; girth 5¾"; weight 4 lbs-10 oz; taken.—

Locality. All over S.P.; T.T.; KIGOMA; MPANDA.
KIMANA, K.C.; KISUMU, K.C.; NAIROBI, K.C.; GALANA R., KILIFI, K.C.; SIGOR, WEST SUK, KENYA; LOKORIS ISLAND, LAKE BARINGO, KENYA; KERIO VALLEY, KENYA.

Habitat. Savanna; Riverine Forest; Coastal thickets.

Altitude. KISUMU = 4400'. They probably do not occur at much higher altitudes than this, but they occur at KISUMU.

Habits. At least partially diurnal. Fairly arboreal but more terrestrial than D. ANGUSTICEPS and D. J. KAIMOSAE. Lie up in hollow trees, disused bee-hives, termite nests, anthear holes, rat holes etc. May be found in large leafy trees. Occasionally enters houses. They may

remain in a hole for a long period
was alarmed & took refuge in a dis
was immediately burnt. No track
at about 4 P.M. on the 15/6/56.— They
resent interference on occasion the
They show anger by opening the
spreading a modified hood and
also hiss loudly when angry.—
off, but, when followed, she
hood, then advanced to a patch
grass for a short time while we
was followed and caught without
motor road which she was cros
a very short distance towards
As soon as I gripped her with
KATABI, BANGWE, KIGOMA; large adu
taken refuge, at about 4 P.M.; —
12.45 P.M., spread a modified hoo
emerging from a hole. She was
N.H. HAJE had been dug out of
species were found just outside the
time. Tracks showed that the
of 30-31/8/64. The hole was bei
seen to have entered the hole,
9.30 A.M. A search was then
a small hole very close to
The hole was dug and a large
of yet another big snake were se
♀ N.H. HAJE & the two ♀♀ D. P. POLYLE
dug out and taken at about 3.
N.H. HAJE and at least 2 adult
in a hollow tree, the snake is
with sticks, and normally does
When disturbed, it usually

)(KIGOMA) (LUO) (MASAI) (SUK) (TUGEN)
GOBOKO; RUWERO; ALASURME; MAENDON, KIPLOGOYON.

. Brown-Mamba, Southern Black-Mamba.
[illegible] flat), straight-sided and moderately
[illegible] round pupil; body relatively slender, though
[illegible]mens, and tapering towards head & tail;
[illegible] anal divided; fangs very far forward in
[illegible] absent; scales smooth & oblique, having
[illegible] anterior suboculars below them, which
[illegible]bial; 2 or 3 postoculars and 2 posterior
[illegible] with the outer border of the parietal;
[illegible] differs in having 2nd upper labial small & not in
[illegible] 25 instead of 23).
[illegible]ly darker on the back than on the sides;
[illegible]tly barred with darker brown; under
[illegible] greyish green above, lighter below.
[illegible] bluish black.

but 7'-6" to 9' is normal in East Africa
-5/4/61; VOI, KENYA; ♂ about 10'-6" (3200 M.M.)
[illegible]"; girth 5⅜"; weight 4 lbs-10 oz; taken —

[illegible] C.; SIGOR, WEST SUK, KENYA; LOKORAE ISLAND, LAKE BARINGO, KENYA;

[illegible].

[illegible] at much higher altitudes than this, but

[illegible] but more terrestrial than D. ANGUSTICEPS
[illegible] — bush, termite nests, earth holes,
. occasionally enters houses. They may

remain in a hole for a long period without emerging — e.g. 13/6/56; MICHAMO MPANDA; adult ♀ was alarmed & took refuge in a disused termites' nest at about midday. The grass round the hole was immediately burnt. No track was found in the ash until she emerged and was taken at about 4 P.M. on the 15/6/56. — They are not normally aggressive, but are bolder and readier to resent interference on occasion than D. ANGUSTICEPS or D. J. KAIMOSAE, particularly when mating. They show anger by opening the mouth wide and shaking the head from side to side, by spreading a modified hood and by protruding the tongue and agitating the tip. They may also hiss loudly when angry. — 5/1/53; NGURUMAHIGA, LIWALE; adult ♀ when alarmed made off, but, when followed, she turned round, reared up about 2', spread a modified hood, then advanced to a patch of longer grass between us, stared at us over the top of the grass for a short time while we remained still, then turned and slowly made off, when she was followed and caught without incident. — 23/4/57; MAKATA, LIWALE; adult ♀ was met on motor road which she was crossing at about 6 P.M.; she reared up about 2', advanced a very short distance towards us, then turned slowly aside into the grass by the roadside. As soon as I gripped her with the stick, she turned straight round on me. — 12/4/56; KATARI, BANGWE, KIGOMA; large adult ♀ (9'-7") taken in the roof inside a hut, in which she had taken refuge, at about 4 P.M. — 19/8/64; KISUMU, KENYA; adult ♂, taken in a [illegible] tree at about 12.45 P.M., spread a modified hood after being caught. — 31/8/64; KIMANA, KENYA; adult ♀ taken emerging from a hole. She was gorged and she spread a modified hood when taken. Adult ♀ N. H. HAJE had been dug out of part of the same hole on the 29/8/64. Old sloughed skins of both species were found just outside the hole showing that both species had occupied it for some time. Tracks showed that the Mamba had entered the hole from outside during the night of 30-31/8/64. The hole was visited daily until on the 6/9/64 tracks of a big snake were seen to have entered the hole, and an adult ♀ D. P. POLYLEPIS was dug out & taken at about 9.30 A.M. A search was then made in the area when a large snake was seen to enter a small hole very close to the hole from which the Mamba & Cobra had been dug. The hole was dug and a large adult ♀ D. P. POLYLEPIS was taken at about 10.30 A.M. Tracks of yet another big snake were seen in the afternoon at the edge of the hole from which the ♀ N. H. HAJE & the two ♀♀ D. P. POLYLEPIS had already been taken, and an adult ♀ N. H. HAJE was dug out and taken at about 3.45 P.M., 6/9/64. It would thus appear that 2 adult ♀♀ N. H. HAJE and at least 2 adult ♀♀ D. P. POLYLEPIS had been using the same hole. — When in a hollow tree, the snake is usually very reluctant to emerge, even when poked with sticks, and normally does so only when its position there becomes untenable. — When disturbed, it usually makes straight for a hole, either in a hollow tree or

[illegible]n the ground, and often descends from a tree to do so, though, if no hole is there, it sometimes climbs to the top of a tree. It descends to the ground more readily than <u>D. ANGUSTICEPS</u> or <u>D. J. KAIMOSAE</u>, and large adults seem to be more terrestrial than the younger specimens.—

<u>[illegible]ison</u>.—June 1949; near MASASI BOMA; a man was bitten in the thumb by both fangs of adult ♀ after she had been pinned down by sticks for several minutes, during which time she had repeatedly bitten the sticks. He was injected with 40 c.c. of polyvalent antivenene made by S.A.I.M.R., JOHANNESBURG within about ten minutes of receiving the bite. He suffered from nothing but a badly swollen hand, and was able to carry a load on the 3rd day after the bite. The swelling subsided after a few days and there were no further ill effects.—

The venom is a powerful neurotoxin.—

<u>Parasites</u>.—22/10/59; SIGOR, WEST SUK, [illegible]

<u>Breeding</u>. Oviparous.—10/2/58; [illegible]

<u>Injuries</u>. Tails are sometimes [illegible]
about one inch from vent.—21/5/[illegible]

<u>Food</u>. Birds.—e.g. 3/7/49; LUM[illegible]
a dash at a small fledgeling [illegible]
the grass on the roof of a gra[illegible]
—8/9/67; ROGOS, LAKE BARINGO, KENYA, [illegible]
a thicket at about midday [illegible]

Mammals.—e.g. 18/7/62; [illegible]
having just swallowed a bat [illegible]

Lizards.—e.g. August, 194[illegible]
stomach.—

[illegible], if no hole is there, it
[illegible]ound more readily than
[illegible]re terrestrial than the

[illegible]y both fangs of adult ♀ after
[illegible] time she had repeatedly
[illegible]were made by S.A.I.M.R.,
[illegible]uffered from nothing
[illegible]the 3rd day after the bite
[illegible] ill effects. –

Parasites. – 22/10/59; SIGOR, WEST SUK, KENYA; adult ♂ had 3 ticks (APONOMMA LATUM) on him. –

Breeding. Oviparous. – 10/2/58; MTENE, RONDO PLATEAU, LINDI; ♀ (7955); length 659 (521+138) M.M. –

Injuries. Tails are sometimes truncated. – e.g. 8/9/64; KIMANA, KENYA; fair sized ♀ had tail truncated about one inch from vent. – 21/8/65; BARWESSA, KERIO VALLEY, KENYA; adult ♂ had truncated tail. –

Food. Birds. – e.g. 3/7/49; LUMESULE, MASASI; adult ♂ descended from mango tree at about midday & made a dash at a small fledgeling chicken, but, on the approach of an African, made off & took refuge in the grass on the roof of a granary from which he was dislodged with a long pole and taken. – – 8/9/67; ROGOS, LAKE BARINGO, KENYA; juvenile had recently swallowed fledgeling bird when taken in a thicket at about midday. Was damaged when taken and had to be destroyed. –

Mammals. – e.g. 19/7/62; GALANA RIVER, KILIFI, K.C.; adult ♂ taken very low down in small tree having just swallowed a bat (LAVIA FRONS REX). –

Lizards. – e.g. August, 1948; near LIHANGWA, LIWALE; adult ♀ had partially digested lizard in stomach. –

adult ♂; PUMUNDA, KILWA; July 1945. Noosed on emerging from hole from which it was driven out, in the morning.
adult ♀; LIWALE BOMA, LIWALE; November 1945. Noosed in a shallow hole in the evening. End of tail truncated.
adult ♀; CORYNDON MUSEUM, NAIROBI, K.C.; 12/2/46. Dug out of rat hole & noosed in the early morning.
adult ♀; LIHANGWA, LIWALE; August 1948. Taken on the move in grass in the afternoon.
adult ♂; TUNDURU DISTRICT; October 1948. Smoked out of hollow tree & noosed in the morning.
adult ♀; NAINOKWE, KILWA; February 1949. Taken on the move after sundown.
adult ♀; about 12 miles West of MASASI BOMA; June 1949. Pelted out of tree with sticks & taken on ground in afternoon.
adult ♂; LUMESULE, MASASI; 3/7/49. Dislodged from granary roof with long pole, taken on ground about midday.
adult ♀; NGURUMBWIGA, LIWALE; 5/1/53. Taken on the move in short grass in the morning.
Half grown ♀; NGOMBIRA, SONGEA; 31/7/53. Taken in grass by roadside in the morning.
Very large adult ♀; MUHURU, MTWARA; 7/3/54. Taken on low branch of tree at about midday.
Large adult ♀; KATABI, BANGWE, KIGAMA; 12/4/56. Taken in hut in roof where she had taken refuge at about 4 P.M.
adult ♀; MICHAMO, MPANDA; 10/6/56. Taken after emerging from hole in which she had stayed 2½ days at about 4 P.M.
adult ♀; MAKATA, LIWALE; 23/9/57. Taken while she was crossing the motor road at about 6 P.M.
Juvenile ♀; MTENE, RONDO, LINDI; 10/2/58. Taken on ground at about 7.30 A.M.
Very large adult ♂; VOI, K.C.; 5/11/61. Taken in a thorn tree at about 10 A.M.
adult ♂; GALANA, KILIFI " 8/7/62. Taken very low down in a small tree at about 11 A.M. Had just eaten L.F.REX.
adult ♂; KIMANA " 9/11/63. Taken in a thorn tree at about 4.30 P.M.
adult ♂; NEWALA BOMA, NEWALA; 7/1/64. Taken in a moderate sized tree at about midday.
adult ♂; KISUMU, KENYA; 18/8/64. Taken in a lowish tree at about 12.45 P.M.
adult ♀; KIMANA " 31/8/64. Taken emerging from hole, out of which N.A.HASE was dug ♀ 29/8/64 at 9.30 A.M.
Young adult ♂ " " 5/9/64. Taken in high thorn tree at about 3.30 P.M.
adult ♀ " " 6/9/64. Dug out of same hole as ♀ 31/8/64 & ♀ N.A.HASE 29/8/64 at about 9.30 A.M.
Large adult ♀ " " " Dug out of small hole at about 10.50 A.M., having been seen to enter it at about 10 A.M.
Fair sized ♀ " " 8/9/64. Taken in a high thorn tree at about 11.30 A.M. Tail truncated close to vent.
Large adult ♀ " " 23/9/64. Taken emerging from hole, which was being dug out, at about 11 A.M.
adult ♂ " " 25/9/64. Taken in a thorn tree at about midday.
adult ♂; SIGOR, WEST SUK, KENYA; 22/10/59. Taken in a high tree at about 8.30 A.M.
adult ♂ " " " " " } Taken close together in a very dense thicket at about 3.15 P.M. and
adult ♂ " " " " " } 3.30 P.M. respectively.
adult ♀; LOKOROS ISLAND, LAKE BARINGO " 25/7/65. Taken under rocks at about 9.50 A.M.
Half grown ♂ " " " " " " Taken in a tree at about 9.55 A.M.
Large adult ♀; BARWESSA, KERIO VALLEY " 31/7/65. Taken under overhanging bank of dry river bed at about 3 P.M.
adult ♂ " " " " Taken very low down in a thicket at about 4.15 P.M.

1/8/65.
adult ♂; BARWESSA, KERIO VALLEY, KENYA. Ta[illegible]
adult ♂ " " " " 2/8/65. Taken on g[illegible]
adult ♂ " " " " 2/8/65. Ta[illegible]
adult ♂ " " " " 3/8/65. Ta[illegible]
adult ♂ " " " " 4/8/65. T[illegible]
adult ♂ " " " " " Ta[illegible]
adult ♂ " " " " 5/8/65. T[illegible]
adult ♂ " " " " 6/8/65. T[illegible]
adult ♂ " " " " 7/8/65. T[illegible]
adult ♀ " " " " 8/8/65. T[illegible]
adult ♂ " " " " 10/8/65. T[illegible]
adult ♂ " " " " " Ta[illegible]
adult ♂ " " " " " Ta[illegible]
adult ♂ " " " " 11/8/65. Du[illegible]
adult ♂ " " " " " Ta[illegible]
adult ♂ " " " " " Ta[illegible]
adult ♂ " " " " 12/8/65. Du[illegible]
adult ♂ " " " " 13/8/65. T[illegible]
Large adult ♂ " " " " 14/8/65. [illegible]
adult ♀ " " " " 15/8/65. Ta[illegible]
Fair sized ♀ " " " " 16/8/65. Ta[illegible]
adult ♀ " " " " 17/8/65. Ta[illegible]
Fair sized ♀ " " " " 18/8/65. T[illegible]
adult ♂ " " " " 19/8/65. Ta[illegible]
adult ♂ " " " " " Ta[illegible]
Fair sized ♀ " " " " 20/8/65. Ta[illegible]
adult ♀ " " " " 21/8/65. Ta[illegible]
adult ♂ " " " " " Ta[illegible]
Fair sized ♀ " " " " Ta[illegible]
adult ♂ " " " " " Ta[illegible]
adult ♀ " " " " 22/8/65. Ta[illegible]
adult ♂ " " " " 24/8/65. Ta[illegible]
adult ♂ " " " " 26/8/65. Ta[illegible]
adult ♀ " " " " " Ta[illegible]

...on which it was driven out, in the morning.
...hole in the morning. End of tail truncated.
...le & noosed in the early morning.
...i grass in the afternoon.
...llow tree & noosed in the morning.
...se after sundown.
...tree with sticks & taken on ground in afternoon.
...with long pole, taken on ground about midday.
...t grass in the morning.
...bush in the morning.
...of tree at about midday.
...roof where she had taken refuge at about [illegible] P.M.
...le in which she had stayed 2½ days at about [illegible] P.M.
...the motor road at about 6 P.M.
...at [illegible]30 A.M.
...at 10 A.M.
...tree at about 11 A.M. Had just eaten L.F. REX.
...[illegible].30 P.M.
...sized tree at about midday.
...t 12.45 P.M.
...which N.R. HASE was dug [illegible] [illegible]/[illegible]/64 at 9.30 A.M.
...t 2.30 P.M.
...N.R. HASE 29/[illegible]/64 at about 9.30 A.M.
...A.M; having been seen to enter it at about [illegible] A.M.
...t 11.30 A.M. Tail truncated close to vent.
...ne being dug out, at about 11 A.M.
...midday.
...[illegible].30 A.M.
...nse thicket at about 3.15 P.M. and
...t 9.50 A.M.
...[illegible] A.M.
...k of dry river bed at about 3 P.M.
...icket at about 4.15 P.M.

adult ♂; BARWESSA, KERIO VALLEY, KENYA. 1/8/65. Taken very low down in a hollow tree at about 3.40 P.M.
adult ♂ " " " 2/8/65. Taken on ground in hollow branch lopped off tree at about 10.45 A.M.
adult ♂ " " " " 2/8/65. Taken in a beehive in a big tree at about 12.35 P.M.
adult ♂ " " " " 3/8/65. Taken very low down in a hollow tree at about 1.55 P.M.
adult ♂ " " " " 4/8/65. Dug out of small hole under a bush at about 12.15 P.M.
adult ♂ " " " " " Taken very low down in a hollow tree at about 4.30 P.M.
adult ♂ " " " " 5/8/65. Taken on ground in hollow branch lopped off tree at about 4.55 P.M.
adult ♂ " " " " 6/8/65. Taken in beehive in large tree at about 3.50 P.M.
adult ♂ " " " " 7/8/65. Taken on ground in hollow branch lopped off tree at about 12.40 P.M.
adult ♀ " " " 8/8/65. Taken in a tree at about 9.25 A.M.
adult ♂ " " " " 10/8/65. Taken in a tree at about 11.40 A.M.
adult ♂ " " " " " Taken in a tree at about midday.
adult ♂ " " " " " Taken in a very dense tree at about 1.50 P.M.
adult ♂ " " " " 11/8/65. Dug out of a hole under a bush at about 12.25 P.M.
adult ♂ " " " " " Taken in a beehive at about 1.25 P.M.
adult ♂ " " " " " Taken in a tree at about 2.55 P.M.
adult ♂ " " " 12/8/65. Dug out of hole in very dense undergrowth at about 2.50 P.M.
adult ♂ " " " " 13/8/65. Taken in a small tree at about 12.15 P.M.
Large adult ♂ " " " " 14/8/65. Taken in dense tree at about 4.15 P.M.
adult ♀ " " " " 15/8/65. Taken on ground in hollow branch lopped from tree at about 12.15 P.M.
Fair sized ♀ " " " " 16/8/65. Taken in very dense high tree at about 2.55 P.M.
adult ♀ " " " 17/8/65. Taken on ground in hollow branch lopped from tree at about 1.55 P.M.
Fair sized ♀ " " " " 18/8/65. Taken in a hollow tree at about 1.35 P.M.
adult ♂ " " " 19/8/65. Taken very low down in hollow tree at about 12.40 P.M.
adult ♂ " " " " " Taken in a beehive in a small tree at about 3.10 P.M.
Fair sized ♀ " " " 20/8/65. Taken very low down in hollow tree at about 10.45 A.M.
adult ♀ " " " " 21/8/65. Taken very low down in a small tree at about 9.55 A.M.
adult ♂ " " " " " Taken in a dense thicket at about 11.45 A.M.
Fair sized ♀ " " " " Taken in a thicket at about 2.05 P.M.
adult ♂ " " " " " Taken in a low tree at about 6.20 P.M.
adult ♀ " " " 22/8/65. Taken on ground in hollow branch lopped from tree at about midday.
adult ♂ " " " " 24/8/65. Taken on move on ground after leaving low bush, at about midday.
adult ♂ " " " 26/8/65. Taken very low down in a thicket at about 11 A.M.
adult ♀ " " " " Taken in a thicket at about 11.15 A.M.

adult ♂; BARWESSA, KERIO VALLEY, KENYA. 27/8/65. Taken in a large hollow tree at about 1:15 P.M.
adult ♀ " " " " 29/8/65. Taken very low down in very dense thicket in narrow gully at about 2 P.M.
adult ♀ " " " " 30/8/65. Taken very low down in a large hollow tree at about 4:05 P.M.
adult ♂ " " " " 31/8/65. Taken in a beehive at about midday.
adult ♀ " " " " 1/9/65. Taken on the ground in dense undergrowth at about 11:10 A.M.
adult ♀ " " " " " Taken very low down in a bush at about 12:15 P.M.
adult ♂ " " " " 3/9/65. Taken very low down in a hollow tree at about midday.
adult ♂ " " " " " Dug out of hole on edge of river bank at about 1:30 P.M.
Half grown ♀ " " " " 6/9/65. Taken in a tree at about 10·10 A.M.
Half grown ♂ " " " 8/9/65. Taken in a high tree at about 9.50 A.M.
adult ♀ " " " " " Taken in a very dense tree at about 3:30 P.M.
adult ♀ " " " " 9/9/65. Taken in same tree as ♂ 3/9/65 at about 10:40 A.M.
adult ♀ " " " " " Taken very low down in a bush at about 1·20 P.M.
adult ♂ " " " " " Taken in a small hole on the edge of a gully at about 2·50 P.M.
adult ♀ " " " " 10/9/65. Taken very low down in a hollow tree at about 5·10 P.M.
adult ♂ " " " " 11/9/65. Dug out of hole among roots of large tree at about 5:40 P.M.
adult ♀ " " " " 12/9/65. Taken out of a beehive at about 11·35 A.M.
adult ♂ " " " " " Taken very low down in a hollow tree at about 12·30 P.M.
adult ♂ " " " " 13/9/65. Taken in a thorn tree at about 10·05 A.M.
Half grown ♀ " " " " 14/9/65. Taken in a small hole on a river bank at about 1·05 P.M.
adult ♀ " " " " " Taken on the ground in hollow branch lopped from a tree at about 1·50 P.M.
Fair sized ♂ " " " " 18/9/65. Taken in a tree at about 6·15 P.M.
Quarter grown ♀ " " " " 20/9/65. Taken in a hollow tree at about 11·25 A.M.
adult ♂ " " " " 21/9/65. Taken in a large hollow tree at about 11·35 A.M.
adult ♂ " " " " " Taken very low down in a thicket at about 12·30 P.M.
adult ♂ " " " " " Dug out of a hole at about 1:15 P.M.
adult ♀ " " " " 24/9/65. Taken low down in a thicket at about 12·50 P.M.
Fair sized ♀ " " " " 25/9/65. Taken on ground in log lopped from tree at about 12:40 P.M.
adult ♂ " " " " " Taken low down in a thicket at about 2·10 P.M.
adult ♀ " " " " 27/9/65. Taken in a tree at about 10·10 A.M.
adult ♂ " " " " " Taken on ground in hollow log lopped from a tree at about 2·50 P.M.
adult ♂ " " " " 29/9/65. Taken very low down in hollow tree at about 2·45 P.M.
adult ♂; RUGUS, LAKE BARINGO " 8/10/65. } Dug out of a disused antbear hole at about 8·29 A.M. and 8·30
adult ♂ " " " " } A.M. respectively.

(Continued on Page 180.)

VIPERA HINDII BOULENGER, 1910.
Description. Snout rounded, with
elliptic pupil; rostral as deep
upper surface of head with in
shield; 10 scales in a transver
labials, 3rd and 4th separated
in contact with the chin shields
S.R. 23-25; V. 124-133; S.C. (paired)

Colour, above, head greyish bro
mark with its point towards th
body greyish brown with four
spots. Below, chin and throat
end of chin to midway to throa

Length up to about 13 inches or a
length 13", tl. 1·35", girth 2·1", weig
length 12½", tl. 1", weight 30 gramm

Locality. ABERDARES, K.C.

Habitat. Moorland above th
ANDROPOGON DUMMERI STAFF.

Habits. Inclined to be sluggish
Were usually found in tussocks
between tussocks when the grou
during sunshine, though occa
was fairly warm. Some were
They inflate the body when a

DENDROASPIS POLYLEPIS POLYLEPIS GÜNTHER, 1864.- Southern Brown-Mamba, Southern Black-Mamba.

(Continued from Page 167).

Specimen	Locality	Date	Remarks
alf grown ♂	Rugus, Lake Baringo, Kenya.	4/10/65.	Taken in a tree at about 10.25 A.M.
adult ♂	„ „ „	„	Taken very low down in a hollow tree at about 11.05 A.M.
adult ♀	„ „ „	6/10/65.	Taken in a tree at about 9.20 A.M.
adult ♂	„ „ „	7/10/65.	Taken in a tree at about 8.35 A.M.
adult ♀	„ „ „	„	Taken in a tree at about 9.05 A.M.
Fair sized ♂	„ „ „	„	Taken very low down in a beehive at about 11.30 A.M.
Fair sized ♂	„ „ „	„	Dug out of a small hole at about midday.
adult ♂	„ „ „	8/10/65.	Taken in a tree at about 10.05 A.M.
adult ♀	„ „ „	„	Taken in a large hollow tree at about 10.55 A.M.
adult ♀	„ „ „	9/10/65.	Taken in a tree at about 9 A.M.
Half grown ♀	„ „ „	„	Taken on the ground in hollow branch lopped off tree at about 10 A.M.
adult ♂	„ „ „	„	Taken in an anthear hole at about 11.40 A.M.
adult ♂	„ „ „	„	Taken in a thicket at about midday.
Large adult ♂	„ „ „	„	Taken in a tree at about 5.30 P.M.
Fair sized ♂	„ „ „	10/10/65.	Taken on mori on ground at about 9 A.M.
adult ♀	„ „ „	„	Taken in a thicket at about 11.30 A.M.
adult ♀	„ „ „	11/10/65.	} ♀ taken entering anthear hole at about 10.15 A.M.; ♀ dug out of
Large adult ♂	„ „ „	„	} same anthear hole at about 10.30 A.M.
Fair sized ♂	„ „ „	„	} Taken under the bark of a dead tree at about 11.30 A.M. and
Juvenile ♀	„ „ „	„	} 11.50 A.M. respectively.
adult ♂	„ „ „	„	Dug out of an anthear hole at about 5.15 P.M.
Large adult ♂	„ „ „	12/10/65.	Taken in an anthear hole at about 9.20 A.M.
Large adult ♂	„ „ „	„	Taken in a very dense thicket at about 9.40 A.M.
Fair sized ♂	„ „ „	„	Taken in a tree at about 10 A.M. Was gorged.
Fair sized ♀	„ „ „	„	Taken in a tree at about 10.45 A.M. Was gorged.
Half grown ♂	„ „ „	„	Taken very low down in a thicket at about midday.
Large adult ♂	„ „ „	„	Taken in a hole at about 6 P.M.
Half grown ♀	„ „ „	„	Taken in a tree at about 6.10 P.M.
Fair sized ♂	„ „ „	13/10/65.	Taken in a thicket at about 8.40 A.M.
adult ♀	„ „ „	„	Taken under rocks at about 9.15 A.M.
Fair sized ♀	„ „ „	„	Taken under the bark of a tree at about 9.50 A.M.
adult ♂	„ „ „	„	Taken under rocks at about 10.05 A.M.
adult ♂	„ „ „	„	Taken in an anthear hole at about 4.10 P.M.
adult ♂	„ „ „	14/10/65.	Taken very low down in a hollow tree at about 10.30 A.M.

Juvenile ♀; RUGUS, LAKE BARINGO, KENYA 14/10/65. Taken in a thicket at about 10.40 A.M.
adult ♂ " " " " " Taken in a thicket at about 11.10 A.M.
adult ♂ " " " " " Taken in a large hole at about 11.50 A.M.
Half grown ♀ " " " " " Taken on ground in hollow branch lopped from tree at about 4.05 P.M.
Quarter grown ♂ " " " 15/10/65. Taken very low down in hollow tree at about 10.15 A.M.
adult ♂ " " " " " Taken on ground under a bush at about 11.40 A.M.
Fair sized ♂ " " " 16/10/65. Taken very low down in a hollow tree at about 11.10 A.M.
Fair sized ♀; SIBILLO, BARINGO " 24/10/65. Taken in a tree at about 11.10 A.M.
adult ♂ " " " " Driven out of a beehive and taken in a high tree at about 1.05 P.M.
adult ♀ " " " " Taken in a tree at about 2.30 P.M.
adult ♂ " " " " Taken in a beehive at about 3.05 P.M.
adult ♂ " " 25/10/65. Taken in a tree at about 9.40 A.M.
adult ♀ " " " " Taken in a beehive at about 1.15 P.M.
Juvenile ♀ " " " Taken on the move on the ground at about 1.45 P.M.
Fair sized ♂ " " 26/10/65. Taken very low down in a thicket at about 10.05 A.M.
Fair sized ♀ " " 27/10/65. Taken in a thicket at about 12.10 P.M.
adult ♂ " " " Taken very low down in a hollow tree at about 2.30 P.M.
adult ♂ " " " Taken very low down in a hollow tree at about 4.20 P.M.
adult ♀ RUGUS, LAKE BARINGO, KENYA 4/9/67. Taken in a tree at about 10.15 A.M.
adult ♂ " " " " Fell out of a tree and taken on the ground at about 10.40 A.M.
adult ♂; " " " " Taken in a tree at about 11.15 A.M.
adult ♀ " " " 5/9/67. Taken in a tree at about 11.25 A.M.
adult ♂ " " " Taken in a tree at about 1 P.M.
adult ♀ " " " 7/9/67. Taken in a thicket at about 9.35 A.M.
adult ♂ " " " " Taken in a tree at about 2.30 P.M.
Fair sized ♀ " " " 8/9/67. Taken in a tree at about 10.30 A.M.
adult ♂ " " " " 11/9/67. Smoked out of a large tree and taken on the ground at about 11.35 A.M.
Half grown ♂ " " " 16/9/67. Taken very low down in a thicket at about 12.40 P.M.
Fair sized ♂ " " " 18/9/67. Taken in the same hollow tree out of which ♂ was smoked 11/9/67 at about [illegible]
Fair sized ♂ " " " 19/9/67. Taken in a tree at about 9.40 A.M.
Fair sized ♂ " " " " Taken in a tree at about 12.50 P.M.
Fair sized ♂ " " " " Taken in a thicket at about 1.35 P.M.
Fair sized ♀ " " " 21/9/67. Taken in a thicket at about midday.
Fair sized ♀ " " " " Taken in a tree at about 1.58 P.M.
Fair sized ♂ " " " 22/9/67. Taken in a tree at about 10 A.M.

Fair sized ♂; RUGUS, LAKE BARINGO, KENYA. 26/9/[illegible]
Juvenile ♂ " " " "
Juvenile ♂ " " " "
adult ♂ " " " 27/9/[illegible]
Fair sized ♀ " "
Fair sized ♂ " " "
adult ♂ " " " 28/9/[illegible]
adult ♂ " " " 29/9/[illegible]
Juvenile ♂ " "
Fair sized ♂ " " " 4/10/[illegible]
adult ♂ " " "
Fair sized ♂ " " " 5/10/[illegible]
Half grown ♂ " " " "
Half grown ♀ " " " 6/10/[illegible]
adult ♂ " " "
Fair sized ♀ " " " "
Fair sized ♀ " " " 7/10/[illegible]
adult ♀ " " "
adult ♂ " " "
adult ♂ " " "
adult ♀ " " "
Fair sized ♀ " " 13/10/[illegible]
adult ♀ " " "
adult ♀ " " "
Juvenile ♂ " " "
adult ♂ " " " 14/1[illegible]
Half grown ♀ " " "
adult ♀ " " " 20/1[illegible]
adult ♂ " " " " 22/1[illegible]
Fair sized ♀; NEVALA BAMA, NEVALA. 15/1[illegible]
Adult ♂; CHESEGON, KERIO VALLEY, KENYA. 13[illegible]
Fair sized ♂ " " 14/7/[illegible]
Adult ♀ " " " "
Adult ♂ " " "

Fair sized ♂; Rugus, Lake Baringo, Kenya. 26/9/67. Taken in a tree at about 12:05 P.M.
Juvenile ♂ " " " " " Taken in a tree at about 12:30 P.M.
Juvenile ♂ " " " " " Taken in a tree at about 3:45 P.M.
adult ♂ " " " " " 27/9/67. Taken in a tree at about 7:45 A.M.
Fair sized ♀ " " " Pelted out of a tree with sticks and taken in a thicket at about 11:35 A.M.
Fair sized ♂ " " " " " Taken in a thicket at about 11:50 A.M.
adult ♂ " " " " " 28/9/67. Taken very low down in a thicket at about 11:40 A.M.
adult ♂ " " " " " 29/9/67. Taken in a thicket at about 8:45 A.M.
Juvenile ♂ " " " " " Taken in a thicket at about 9:25 A.M.
Fair sized ♂ " " " " " 4/10/67. Taken on the ground in an isolated bush at about 10:40 A.M.
adult ♂ " " " " " Taken in a bush at about 10:55 A.M.
Fair sized ♂ " " " " " 5/10/67. Taken very low down in a thicket at about 11:30 A.M.
Half grown ♂ " " " " " Taken in a thicket at about 12:45 P.M.
Half grown ♀ " " " " " 6/10/67. Taken in a tree at about 8:40 A.M.
adult ♂ " " " " " Taken in a thicket at about 11:30 A.M.
Fair sized ♀ " " " " " Taken in a tree at about 12:30 P.M.
Fair sized ♀ " " " " " 7/10/67. Taken in a thicket at about 10:50 A.M.
adult ♀ " " " " " Taken in a thicket at about 11:10 A.M.
adult ♂ " " " " " Taken in a thicket at about 11:30 A.M.
adult ♂ " " " " " Taken in a tree at about 12:55 P.M.
adult ♀ " " " " " Taken in a tree at about 2:05 P.M.
Fair sized ♀ " " " " " 13/10/67. Taken in a tree at about 10 A.M.
adult ♀ " " " " " Taken in a tree at about 10:50 A.M.
adult ♀ " " " " " Taken in a tree at about 11:30 A.M.
Juvenile ♂ " " " " " Taken on the ground out of hollow log at foot of tree at about 2:40 P.M.
adult ♂ " " " " " 14/10/67 Taken in a thicket at about 9:15 A.M.
Half grown ♀ " " " " " Taken in a tree at about 1:15 P.M.
adult ♀ " " " " " 20/10/67. Taken in a thicket at about 12:25 P.M.
adult ♂ " " " " " 22/10/67. Dug out of a hole among roots of tree at about 11:15 A.M.
Fair sized ♀; Nakuru Bana, Nakuru. 15/11/67. Taken very low down in the roof of a hut at about 4:30 P.M.
Adult ♂; Chesegon, Kerio Valley, Kenya. 13/7/68. Taken in a tree at about 4:40 P.M.
Fair sized ♂ " " " 14/7/68. Taken on the ground at about 11:40 A.M.
Adult ♀ " " " " Taken in a high tree at about 2:30 P.M.
Adult ♂ " " " " Taken very low down in a thicket at about 3:55 P.M.

adult ♀, CHESEGON, KERIO VALLEY, KENYA, 15/7/68. Taken low down in a tree at about 4.05 P.M.

" ♂ " " " " " Taken in a low tree at about 10.20 A.M.

Fair sized ♀ " " " " " Dug out of a small hole at about 4.55 P.M.

adult ♀ " " " " 16/7/68. Taken in a dense tree at about 12.45 P.M.

" ♂ " " " " " Taken in a beehive at about 7 P.M.

" ♂ " " " " 18/7/68. Dug out of a hole at about [illegible]:30 A.M.

" ♀ " " " " " Taken under a log at about 12.15 P.M.

Half grown ♂ " " " " 19/7/68. Taken very low down in a dense thorn tree at about [illegible]

Adult ♀ " " " " " Taken in a beehive at about 3.40 P.M.

adult ♂ " " " " " Taken in a hollow log at about 4.50 P.M.

" ♂, MPOPUT " " " 25/7/68. Taken in a tree at about 9.30 A.M.

" ♀ " " " " " Taken in a tree at about 2 P.M.

Fair sized ♂ " " " " 27/7/68. Taken in a solitary tree at about 2.55 P.M.

" ♀ " " " " 28/7/68. Taken in a tree at about 9.30 A.M.

" ♂ " " " " " Taken very low down in a thorny tree at about 6.20 P.M.

" ♂ " " " " 29/7/68. Taken in a thorny tree at about 12.20 P.M.

Half grown ♀ " " " " 31/7/68. Taken in a tree at about 11.30 A.M.

adult ♂ " " " " " Taken on the move on the ground at about 1.15 P.M.

Fair sized ♀ " " " " 1/8/68. Taken very low down in a hollow tree at about 4.50 P.M.

" ♂ " " " " 2/8/68. Dug out of a small hole at about 10 A.M.

Adult ♂ " " " " " Taken very low down in dense tree at about 12.45 P.M.

" ♀ " " " " 3/8/68. Taken in a tree at about 12.30 P.M.

Fair sized ♂ " " " " 5/8/68. Dug out of a hole in a thick hedge at about 10.20 A.M.

...ergrowth or go into a hole
...ves if cornered or molested:
...ay hang on and chew. They
...MACRURUS, 360 (198 + 162) mm;

NAJA MELANOLEUCA HALLOWELL, 1857. — Black-and-White Cobra.

Juvenile ♀; EPULU, ITURI FOREST, BELGIAN CONGO; 17/8/54. Taken in roof of "banda" in afternoon.
Juvenile ♀; MAYALA " " " 3/9/54. Taken in hut in evening.
Adult ♀ " " " " " 6/9/54. Taken in a hut behind a heap of bags of grain in morning.
Juvenile ♂ " " " " " 10/9/54. Taken under a log outside a hut in the morning.
Very large adult ♀; MAKOTE, NEWALA; 10/9/55. Taken in lower branches of tree at about 11 A.M. About 7-[illegible]
Adult ♂; LIWALE BOMA, LIWALE; 11/6/58. Taken in dense thicket at about 1 P.M., by small stream.
Juvenile ♀ " " " 16/11/58. Swam across stream, landed & was taken at foot of tree at about 11 A.M.
Adult ♂; KAKAMEGA FOREST, K.C.; 13/10/59. Dug out of hole in thick undergrowth by roadside at about 12:30 P.M.
Adult ♀; LIWALE BOMA, LIWALE; 6/12/59. Taken in thicket by [illegible] swallowing R.C. MACRURUS at about 5:30 P.M.
Adult ♂; MPULUNGU, N.R.; 1/8/60. Taken on dry land in grass in act of taking refuge in water at about 9:50 A.M.
Fair sized ♀; MWAYA, TUKUYU; 10/8/60. Dug out of small hole under bush, where she had taken refuge, at about 6 P.M.
Adult ♂ " " 19/8/60. Taken in a low dense bushy tree at about 2:15 P.M.
Juvenile ♂ " " 21/8/60. Taken in a low tree at about 4 P.M. He had recently fed.
Juvenile ♀ " " 26/8/60. Dug out of hole in a banana grove at about 3 P.M.
Half grown ♀ " " 2/9/60. Taken in a house at about 4 P.M.
Adult ♂; MIHURU, NEWALA; 11/3/61. Taken in a native house under a metal drum at about 1:45 P.M.
Adult ♂; KAKAMEGA FOREST, K.C.; 9/9/61. Dug out of termites' nest after over two days digging at about 11:30 A.M.
Fair sized ♂ " " " 2/10/61. Taken fairly low down in a tree at about 2:55 P.M.
Young adult ♂ " " " 9/10/61. Taken in a bush as he was making off from roof of a [illegible] at about [illegible]
Fair sized ♀; NEWALA BOMA, NEWALA; 16/5/63. Dug out of small hole where she had taken refuge at about 9 A.M.
Adult ♂; KAKAMEGA FOREST, K.C.; 22/10/63. Dug out of used termites' nest at about 3:30 P.M. About 7'.
Large adult ♂ " " " 24/10/63. Dug out of another part of same termites' nest as ♂ of 22/10/63 at about 9:30 A.M.
Very large adult ♂; NANGURUWE, NEWALA; 3/6/64. Taken high up in a hollow tree at about 12:30 P.M. 8'-8", 7 lbs-10 oz. 7-3" (about 2 hours [illegible])
Large adult ♂; KISUMU, KENYA; 16/7/64. Dug out of hole after descending from tree at about 3:30 P.M. Over 7'.
Fair sized ♂ " " 2/8/64. Dug out of a hole in a bank dividing two rice fields at about midday.
Adult ♂ " " " Taken in very dense tree at about 3:45 P.M.
Adult ♂ " " " Taken in a low tree at about 5:40 P.M.
Adult ♀ " " 10/8/64. Dug out of hole in bank dividing two rice fields at about 2:30 P.M.
Adult ♀ " " 11/8/64. Dug out of a small hole in a dense bush at about 5:30 P.M.
Adult ♂ " " 13/8/64. Taken in low thicket bordering airstrip at about 3:30 P.M.
Half grown ♀ " " 19/8/64. Taken under dead grass between two rice fields at about 3:20 P.M.
Adult ♂; MAKOTE, NEWALA; 29/6/65. Taken coming out of hollow tree at about 3:40 P.M. Trouble with [illegible]
Very large adult ♂; NANGURUWE " 24/2/66. Taken in a cashew tree at about 10:50 A.M.
Large adult ♂ " " 23/7/66. Taken in a mango tree at about 9:30 A.M.

adult ♀; LIDUMBE, NEWALA; 7/4/67. Taken in a very low dense thicket at about 9.55 A.M.

Juvenile ♀; NANGURUWE " 14/7/67. Taken in a hollow, dead, fallen tree at about 10.05 A.M.

BITIS NASICORNIS (SHAW, 1802). —

(Cont

8/10/63; ♀; KAKAMEGA FOREST, K.C. – Juveni

10/10/63; ♀ " " " Large ad

" ♀ " " " Adult

12/10/63; ♂ " " " Juvenil

13/10/63; ♀ " " " Half gr

14/10/63; ♀ " " " adult

19/10/63; ♀ " " " Large ad

22/10/63; ♂ " " " Fair s

30/10/63; ♀ " " Greater gr

[illegible]

Page.

[E]LAPSOIDEA SUNDEVALLII DECOSTERI. — 2.
[E]LAPSOIDEA SUNDEVALLII GÜNTHERI. — 4.
[B]ULENGERINA ANNULATA STORMSI. — 6, 7, 8, 9, 10, 11.
[N]AJA HAJE HAJE. — [illegible] 130, 131, 132.
[N]AJA MELANOLEUCA. — [illegible] 122, 123, 124, 125.
[N]AJA NIGRICOLLIS PALLIDA. — 141, 142.
[N]AJA NIGRICOLLIS NIGRICOLLIS. — [illegible] 148, 149, 150, 151.
[P]SEUDOHAJE GOLDII. — 28, 29.
[D]ENDROASPIS JAMESONI KAIMOSAE. — 152, 153, 154, 155, 156, 157.
[D]ENDROASPIS ANGUSTICEPS. — 158, 159, 160
[D]ENDROASPIS POLYLEPIS POLYLEPIS. — 161, 162, 163, 164, 165, 166, 167, 180.
[P]ELAMIS PLATURUS. — 42, 43.
[A]TRACTASPIS IRREGULARIS BIPOSTOCULARIS. — 53.
[A]TRACTASPIS MICROLEPIDOTA MICROLEPIDOTA — 51.
[A]TRACTASPIS CORPULENTA. — 46.
[A]TRACTASPIS BIBRONII ROSTRATA. — 48, 49, 50.
[C]AUSUS RHOMBEATUS. — 54, 55.
[C]AUSUS RESIMUS. — 56.
[C]AUSUS DEFILIPPII. — 58, 59.
[C]AUSUS LICHTENSTEINII. — 57.
[V]IPERA HINDII. — 172, 173, 174, 175, 176, 177, 178.
[V]IPERA SUPERCILIARIS — 129.
[illegible] WORTHINGTONI. — 68, 69.
[B]ITIS ARIETANS ARIETANS. — 70, 71.
[B]ITIS GABONICA GABONICA. — 74, 75, 76.
[B]ITIS NASICORNIS. — 94, 95, 96, 97, 128.
[E]CHIS CARINATUS PYRAMIDUM. — 109, 110, 111, 112, 113, 114, 115, 116, 117, 118, 119, 120, 121.
[A]THERIS SQUAMIGER. — 102, 103, 104.
[A]THERIS NITSCHEI NITSCHEI. — 106, 107.
[E]CHIS COLORATUS — 108,
[C]ERASTES CERASTES — 183.
[A]THERIS DESAIXI — [illegible] 140.

[P]ERSONAL INCIDENTS WITH SNAKES. — 185.

ELAPSOIDEA SUNDEVALLII DEC[illegible]

Description: S.R. 13; V. [illegible] 175; S.C[illegible]
flat [illegible] slightly distinct from [illegible]
robust; tail very short and [illegible]
Colour of adults may be uniform [illegible]
visible, particularly when the body [illegible]
black, or whitish. Juveniles have [illegible]
or narrower white bands; [illegible]
Length up to about 2 feet.

Locality. All over S.P., T.T. [illegible]

Habitat. Savanna.

Habits. Mainly nocturnal. A bur[illegible]
under rotting rubbish, fallen lo[illegible]
Inflates it's body when angry.

Food. Amphibians. — e.g. H.M.MAR[illegible]

Snakes. — e.g. M.S. SEMIOR[illegible]

Breeding. — 31/1/52; LIWALE [illegible] 4 f[illegible]
[illegible] hatched juveniles taken in [illegible]

Poison. A neurotoxin.

SNAKES.

…[illegible] SNAKES.)

' venom into right eye. Washed it with milk.
t native "dawa". No treatment.
fang. No treatment.
g. No treatment.
eye. Washed it with weak permanganate.
s. No treatment.
No treatment.
treatment.
treatment.
g. No treatment.
No treatment.
ft eye. Washed it with water.
ngs - 30 c.c. Serum injected.

POISONOUS FRONT-FANGED SNAKES FROM 1/1/45.

	31/12/62.	31/12/63.	31/12/64.	31/12/65.	31/12/66.	31/12/67.
B. FASCIATUS	-	-	-	-	1.-	1.-
E.B. GÜNTHERI	6.-	6.-	6.-	6.-	6.-	6.-
E.S. DECOSTERI	22.-	22.-	22.-	22.-	22.-	22.-
D.A. STORMSI	75.-	75.-	75.-	75.-	75.-	75.-
N.H. HAJE	3.-	7.-	11.-	11.-	11.-	11.-
N. MELANOLEUCA	19.-	22.-	31.-	32.-	34.-	36.-
N.N. PALLIDA	2.-	2.-	2.-	2.-	2.-	2.-
N.N. NIGRICOLLIS	56.-	97.-	139.-	195.-	259.-	386.-
P. GOLDII	1.-	1.-	1.-	1.-	1.-	1.-
D.J. KAIMOSAE	54.-	77.-	77.-	77.-	77.-	77.-
D. ANGUSTICEPS	1277.-	1847.-	2786.-	3366.-	6005.-	1633.-
O. HANNAH	-	-	-	-	16.-	16.-
N.N. KAOUTHIA	-	-	-	-	5.-	5.-
D.P. POLYLEPIS	20.-	27.-	30.-	154.-	154.-	201.-
P. PLATURUS	1.-	1.-	1.-	1.-	1.-	1.-
A.M. MICROLEPIDOTA	2.-	2.-	2.-	2.-	2.-	2.-
A. CORPULENTA	1.-	1.-	1.-	1.-	1.-	1.-
A.B. ROSTRATA	45.-	48.-	49.-	49.-	50.-	50.-
A.I. BIPOSTOCULARIS	-	-	2.-	2.-	2.-	2.-
V.R. SIAMENSIS	-	-	-	-	1.-	1.-
C. RHOMBEATUS	35.-	35.-	36.-	36.-	37.-	37.-
C. RESIMUS	1.-	1.-	2.-	2.-	2.-	2.-
C. DEFILIPPII	221.-	233.-	265.-	277.-	288.-	314.-
C. LICHTENSTEINII	1.-	1.-	1.-	1.-	1.-	1.-
V. HINDII	74.-	74.-	118.-	122.-	122.-	122.-
C. CERASTES	-	-	-	10.-	10.-	10.-
B. WORTHINGTONI	7.-	7.-	7.-	7.-	7.-	7.-
B.A. ARIETANS	203.-	246.-	290.-	314.-	330.-	354.-
B.G. GABONICA	1057.-	1260.-	1553.-	1757.-	1974.-	2159.-
B. NASICORNIS	62.-	77.-	77.-	77.-	77.-	77.-
E.C. PYRAMIDUM	312.-	312.-	312.-	312.-	312.-	312.-
A. SQUAMIGER	21.-	29.-	29.-	29.-	29.-	29.-
A.N. NITSCHEI	[illegible]	[illegible]	[illegible]	[illegible]	1.-	1.-
E. COLORATUS	-	-	-	2.-	2.-	2.-
A. RHODOSTOMA	-	-	-	-	8.-	8.-

[illegible]

PERSONAL INCIDENTS WITH SNAKES.

(FOR DETAILS, SEE NOTES ON THE INDIVIDUAL SNAKES.)

August 1942; MANDERA, BRITISH SOMALILAND; N.N.N. sprayed venom into right eye. Washed it with milk.
April 1945; KIPILIPILI, LIWALE; C.D. made to bite 4 times to test native "[illegible]". No treatment.
February 1948; RUPONDA, NACHINGWEA. B.A.A. bit with one fang. No treatment.
30/12/51; NAMBUNJU, TUNDURU. A.B.R. jabbed with one fang. No treatment.
19/7/54; LIWALE BOMA. N.N.N. sprayed venom into left eye. Washed it with weak permanganate.
22/4/59; LIWALE BOMA. A.B.R. jabbed with both fangs. No treatment.
24/3/60; MIHORO, NEWALA. C.D. bit with both fangs. No treatment.
14/4/60; NEWALA BOMA. D.T. bit with both fangs. No treatment.
17/12/60; NEWALA BOMA. D.T. bit with both fangs. No treatment.
30/12/61; NAMBUNGA, NEWALA. B.A.A. bit with one fang. No treatment.
3/3/63; NEWALA BOMA. D.T. bit with both fangs. No treatment.
[illegible]/1/64; NEWALA BOMA. N.N.N. sprayed venom into left eye. Washed it with water.
29/2/64; NANGURUWE, NEWALA. D.A. bit with both fangs. 30 c.c. Serum injected.

Perso[illegible]

	31/12/62	31/12/63	31/12/[illegible]
D. FASCIATUS	–	–	–
E. S. GÜNTHERI	6	6	6
E. S. DECOSTERI	22	22	22
B. A. STORMSI	75	75	75
N. H. HAJE	3	7	11
N. MELANOLEUCA	19	22	31
N. M. PALLIDA	2	2	2
N. N. NIGRICOLLIS	56	97	139
P. GOLDII	1	1	1
D. J. KAIMOSAE	54	77	77
D. ANGUSTICEPS	1277	1847	2706
O. HANNAH	–	–	–
N. N. KAOUTHIA	–	–	–
D. P. POLYLEPIS	20	24	30
P. PLATURUS	1	1	1
A. M. MICROLEPIDOTA	2	2	2
A. CORPULENTA	1	1	1
A. B. ROSTRATA	45	48	49
A. J. BIPOSTOCULARIS	–	–	2
V. R. SIAMENSIS	–	–	–
C. RHOMBEATUS	35	35	36
C. RESIMUS	1	1	2
C. DEFILIPPII	221	233	265
C. LICHTENSTEINII	1	1	1
V. HINDII	74	74	118
C. CERASTES	–	–	–
B. WORTHINGTONI	7	7	7
B. A. ARIETANS	203	246	290
B. G. GABONICA	1057	1260	1553
B. NASICORNIS	62	77	77
E. C. PYRAMIDUM	312	312	312
A. SQUAMIGER	21	29	29
A. V. NITSCHEI	1	1	1
E. COLORATUS	–	–	–
A. RHODOSTOMA	–	–	–
TOTAL	3579	4505	5926

123, 124, 125.
42.
149, 150, 151.

152, 153, 154, 155, 156, 157.

161, 162, 163, 164, 165, 166, 167, 180.

5, 116, 117, 118, 119, 120, 121.

(NGINDE)
Local Name – LISOKA USHANGA.

ELAPSOIDEA SUNDEVALLII DECOSTERI BOULENGER, 1888. – Southeastern Garter Snake.

Description. S.R. 13; V. less than 175; S.C. (paired) less than 30; loreal absent; head short, broad, flat & only slightly distinct from neck; snout broader than long; eye small with round pupil; body fairly robust; tail very short and ending in a spike. V. 137-169; S.C. (paired) 13-29; anal entire.

Colour of adults may be uniformly black above, though usually faint white bands are visible, particularly when the body is inflated in anger; underparts may be plumbeous, black, or whitish. Juveniles have a grey head; body above is banded alternate broad black or narrower white bands; underparts whitish.

Length up to about 2 feet.

Locality. All over S.P., T.T.

Habitat. Savanna.

Habits. Mainly nocturnal. A burrowing species usually found in wet season. Lies up under rotting rubbish, fallen logs etc. Quick & active. Gentle and easily [illegible]. Inflates its body when angry.

Food. Amphibians – e.g. H. M. MARMORATUS; A. S. WHYTII.

Snakes. – e.g. M. S. SEMIORNATUS, T. S. MUCRUSO.

Breeding. – 31/1/52; LIWALE [illegible] 4 fully developed eggs. – Eggs are large & cylindrical. – Newly hatched juveniles taken in June, S.P., T.T. –

Poison. A neurotoxin.

ELAPSOIDEA SUNDEVALLII GÜNTHERI Bocage, 1866. – Western Garter – Snake.

Description. Very similar in appearance to E. S. DECOSTERI. Head short, broad, flat, and only slightly distinct from neck; eye small, with round pupil; body cylindrical; S.C. paired; tail very short ending in a spike; V. 138–167; S.C. 13–26; snout rounded; anal entire.

Colour of adult, above, black with a series of narrow white bands across back & tail; chin and throat white; anterior ventrals may be white-edged, remainder of underside black.
Length up to over 25 inches.

Locality. KAKAMEGA, K.C.

Habitat. Forest and its vicinity.

Habits. Similar to those of E. S. DECOSTERI.

Food. Amphibians.

Reptiles – e.g. small snakes.

Poison. A neurotoxin.

Zo9269; ♀; KAKAMEGA, K.C.; 21/9/61; length 591 (548+43) M.M.; Found dead on edge of thicket at about 10.30 A.M.
Large adult ♂ " " 30/9/61 – 636 (579+57) M.M.; about 3/4–1 1/2"; Taken in small clump of high grass about [illegible]
Adult ♀ " " 1/10/61; Taken in short grass at about 9.30 A.M.
Half grown ♀ " " 3/10/61; Taken in undergrowth of old shamba at about 10.50 A.M.
Adult ♀ " " 20/10/61; Taken in a new latrine pit at about 10 A.M.
Large adult ♂ " " 22/10/61; Dug out from among tree roots at about 10.15 A.M.

Local Names. — MWIU (MPULUNGU); KIROBA (KIGOMA).

BOULENGERINA ANNULATA STORMSI Dollo, 1886. — Tanganyika Water Cobra.

Description. S.R. 21-23; V. 192-211; S.C. (paired) 67-78; anal entire; Scales smooth and glossy; eye moderate; head short, broad, deep, and not very distinct from the neck which can be flattened and expanded into a hood which is not so pronounced as that of cobras of the genus NAJA; body of adults thick & heavy; tail moderate or shortish — e.g. 20/8/58; MPULUNGU, N.R.; ♀; length 7'-3", tail 1'-4" (i.e. about 5.4 times into total length). Pupil round.

Colour, above, russet to dark brown anteriorly becoming darker posteriorly. There is a number of black bars across neck & anterior part of body some of which may form complete rings. Underparts white anteriorly becoming progressively darker until posterior portion of body below and tail are black.

Length up to over 8 feet. — 20/8/58; MPULUNGU, N.R.; ♀; length 7'-4" (about 2235 M.M.), girth 6" (had just laid 22 eggs), weight 7 lbs. — 21/8/58; MPULUNGU, N.R.; ♀; length 7'-1", girth 8½", weight 8 lbs. — 27/7/60; MPULUNGU, N.R.; 2 ♀♀ weighing 8½ lbs & 9 lbs respectively taken at about 9 A.M. & 3.45 P.M. —

Locality. KIGOMA; KAREMA, MPANDA.
MPULUNGU, N.R.

Habitat: Vicinity of rocky shores of LAKE TANGANYIKA.

Habits. Aquatic. Both diurnal & nocturnal. They occasionally sun themselves on the rocks just after sunrise or late in the evenings. They lie up in crevices among the rocks at about waterline (The crevices among submerged sandbags against "LIEMBA" pier were favourite haunts at KIGOMA). At MPULUNGU some were seen daily around the submerged rocks at the "LIEMBA" pier, leaving or entering the crevices among the rocks & occasionally basking on the rocks only partially submerged. At KIGOMA, where they were not common, it was noticed that one would take up its abode for several days in one place, leaving & returning to it, & then move on elsewhere. When in the water they seem to spend most of their time well beneath the surface, only coming up occasionally to breathe. They seem to be shy & not aggressive, as the locals bathe fearlessly in the vicinity of their haunts. When caught, they almost always demonstrated with spread hoods and wide open mouths. They never sprayed their venom

and seem unable to do so. They are very powerful, being difficult to dislodge from the crevices in the rocks, particular if the posterior part of the body is caught & the anterior part gets wedged into a crevices. Specimens sent to the LONDON ZOO became mainly terrestrial in captivity.

Poison: a powerful neurotoxin.

Parasites:- 21/8/58; MPULUNGU, N.R.; adult ♀ had APONOMMA LATUM on head.
- 26/7/60 " " large adult ♀ " APONOMMA LATUM " "
- 30/7/60 " " adult ♀ " 3 APONOMMA LATUM " "

Breeding: Oviparous. — September 1958, large adult ♀, taken MPULUNGU, N.R., 17/8/58, laid eggs in CHICAGO ZOO, BROOKFIELD PARK. — 11/9/58, large adult ♀, taken MPULUNGU, N.R., 20/8/58, laid 22 eggs in CORYNDON MUSEUM, NAIROBI. — August 1960, adult ♀, taken MPULUNGU, N.R., 30/7/60, laid 23 eggs in NEW YORK ZOO. — End of August 1960, adult ♀, taken MPULUNGU, N.R., 1/8/60 laid 22 eggs of which 15 were fertile in PORT ELIZABETH SNAKE PARK, S.A. — 28/6/56; KAREMA, MPANDA; ♀ 624 (501+123) M.M. — 5/7/56; KAREMA, MPANDA; ♂ 486 (394+92) M.M, and ♀ 646 (521+125) M.M. —

Food: Fish. — e.g. 23/5/56; KALALANGABO, KIGOMA; adult ♀ seen by me from canoe swimming towards a sobbing rather flat-topped rock, her head raised slightly above surface, holding a large fish by the head in her mouth and propelling it in front of her. She reached the rock, came out on to it with the fish & began to swallow it. I then came up & caught her when she disgorged the fish. When I returned to look for the fish, after rowing ashore & bagging the snake, it had disappeared. —

Total up to 3/8/60. — 75 (26 ♂♂, 49 ♀♀).

Amphibians. — e.g. Captive specimens in LONDON ZOO were fed on frogs. —

20/4/56; adult ♀; KIGOMA BOMA. Taken under lower platform of Power House Pier at about 9.30 A.M.
22/5/56; adult ♀; KALALANGABO, KIGOMA. Taken on a rock as she was swallowing a large fish at about 9.30 A.M.
25/5/56; adult ♂; KIGOMA BOMA. Taken in deep water by "LIEMBA" pier emerging from submerged sand bags 5.30 P.M.
1/6/56; fair sized ♂ " " Taken on emerging from crevice in same submerged sandbags about 6 P.M.
2/6/56; adult ♂ " " Taken as his head emerged from hole in concrete in very shallow water 6 P.M.
28/6/56; juvenile ♀; KAREMA, MPANDA. Taken with a hand net in water from canoe near rocky shore 9 A.M.;
5/7/56; juvenile ♂ " " " " " " " " " " " " " " 9.30 A.M.;
" juvenile ♀ " " " " " " " " " " " " " 10 A.M.;
16/8/58; adult ♀; MPULUNGU, N.R. Taken among rocks in shallow water at about 5 P.M.
17/8/58; adult ♀ " " " " " " " " " " 5 P.M.
" adult ♀ " " " " " " " " " " 5.15 P.M.
" adult ♀ " " " " " " " " " " 5.30 P.M.
" Very large adult ♀ " " " " " " " " " 6 P.M.
18/8/58; Very large adult ♂ " " Taken basking on rocks half out of the water at about 7.30 A.M.
" juvenile ♀ " " Taken among rocks in shallow water at about 4.30 P.M.
" adult ♂ " " " " " " " " " " 5 P.M.
" Large adult ♂ " " " " " " " " " " 5.15 P.M.
" Large adult ♂ " " " " " " " " " " 5.30 P.M.
20/8/58; Very large adult ♀ " " " " " " " " " " 4.30 P.M.
" adult ♂ " " " " " " " " " " 4.45 P.M.
" adult ♂ " " " " " " " " " " 5 P.M.
" Very large adult ♀ " " " " " " " " " 6 P.M.
21/8/58; Very large adult ♀ " " " " " " " " " 4.30 P.M.
" Very large adult ♀ " " " " " " " " " 5 P.M.
" Large adult ♀ " " " " " " " " " " 5.15 P.M.
" Adult ♀ " " " " " " " " " " 5.30 P.M.
23/8/58; adult ♂ " " " " " " " " " 5.30 P.M.
24/8/58; adult ♂ " " " " " " " " " 5 P.M. Damaged by rocks.
26/8/58; Large adult ♂ " " " " " " " " " 5 P.M.
" adult ♂ " " Taken basking on rocks half out of the water at about 5.30 P.M.
27/8/58; Large adult ♂ " " Taken in shallow water near the rocks at about 4.30 P.M.
" Large adult ♂ " " " " " " " " " " 5 P.M.
" Large adult ♀ " " " " " " " " " " 5.30 P.M.
22/7/60; adult ♀ " " Taken among rocks barely submerged at about 3.40 P.M.

22/7/60; Large adult ♀; MPULUNGU, N.R.
24/7/60; adult ♀ " " Ta
" Fair sized ♂ " " "
" Half grown ♀ " " Tak
" Half grown ♀ " " Take
" Large adult ♀ " " Take
25/7/60; Juvenile ♀ " " Take
" Large adult ♀ " " "
" Adult ♂ " " "
" Large adult ♀ " " "
26/7/60; Half grown ♀ " " Taken
" Large adult ♀ " " Taken
" Large adult ♀ " " Taken
27/7/60; Very large adult ♀ " " Taken
" Adult ♀ " " Taken
" Large adult ♀ " " Taken
" Large adult ♀ " " "
" Adult ♀ " " "
" Very large adult ♀ " " Taken
" Adult ♂ " " Taken
" adult ♀ " " "
" adult ♀ " " "
28/7/60; Adult ♂ " " "
" Adult ♀ " " "
" Very large adult ♀ " " Taken
" Adult ♂ " " Taken
30/7/60; Large adult ♀ " " "
" Large adult ♀ " " "
" Half grown ♀ " " Taken in
" Very large adult ♀ " " Taken
" Adult ♂ " " "
31/7/60; Adult ♂ " " Taken
" Half grown ♀ " " Taken
1/8/60; adult ♀ " " "

…ower House Pier at about 9.30 A.M.
…swallowing a large fish at about 9.30 A.M.
…seen emerging from submerged sand bags 5.30 P.M.;
Same submerged sandbags about 6 P.M.
…hole in concrete in very shallow water 6 P.M.;
…from canoe near rocky shore 9 A.M.;
" " " " " 9.30 A.M.;
" " " " " 10 A.M.;
water at about 5 P.M.
" " " 5 P.M.
" " " 5.15 P.M.
" " " 5.30 P.M.
" " " 6 P.M.
…f the water at about 7.30 A.M.
…water at about 4.30 P.M.
" " " 5 P.M.
" " " 5.15 P.M.
" " " 5.30 P.M.
" " " 4.30 P.M.
" " " 4.45 P.M.
" " " 5 P.M.
" " " 6 P.M.
" " " 4.30 P.M.
" " " 5 P.M.
" " " 5.15 P.M.
" " " 5.30 P.M.
" " " 5.30 P.M.
" " " 5 P.M. Damaged by rocks.
" " " 5 P.M.
water at about 5.30 P.M.
…rocks at about 4.30 P.M.
" " " 5 P.M.
" " " 5.30 P.M.
…d at about 3.40 P.M.

22/7/60;	Large adult ♀;	MPULUNGU, N.R.	Taken in shallow water after struggle to extract her from rocks at about [illegible] P.M.
24/7/60;	adult ♀	"	" Taken among rocks in shallow water at about 7.30 A.M.
"	Fair sized ♂	"	" " " " " " " " " " 1.30 P.M.
"	Half grown ♀	"	" Taken in fairly shallow water coming in from open lake at about 2.45 P.M.
"	Half grown ♀	"	" Taken with short tongs edge of hole in concrete of small pier at about 3.30 P.M.
"	Large adult ♀	"	" Taken among rocks in fairly shallow water at about 3.45 P.M.
25/7/60;	Juvenile ♀	"	" Taken among rocks in shallow water at about 7.45 A.M.
"	Large adult ♀;	"	" " " " " " " " " " 11.45 A.M.
"	Adult ♂	"	" " " " " " " " " " 1.15 P.M.
"	Large adult ♀	"	" " " " " " " " " " 2.15 P.M.
26/7/60;	Half grown ♀	"	" Taken among rocks in very shallow water at about 8 A.M.
"	Large adult ♀	"	" Taken emerging from rocks after a long wait at 3 P.M. Had several wounds on her.
"	Large adult ♀	"	" Taken among rocks barely submerged at about 4.15 P.M.
27/7/60;	Very large adult ♀	"	" Taken when entering crevice among rocks after hard struggle at about 9 A.M.
"	Adult ♀	"	" Taken among rocks in rather deep water at about 12.45 P.M.
"	Large adult ♀	"	" Taken among rocks in shallow water at about 1 P.M.
"	Large adult ♀	"	" " " " " " " " " " 1.50 P.M.
"	Adult ♀	"	" " " " " " " " " " 3.30 P.M.
"	Very large adult ♀	"	" Taken while entering a crevice in rocks after very hard struggle at about 3.45 P.M.
"	Adult ♂	"	" Taken among rocks in shallow water at about 4.30 P.M.
"	Adult ♀	"	" " " " " " " " " " 4.40 P.M.
"	Adult ♀	"	" " " " " " " " " " 5 P.M.
28/7/60;	Adult ♂	"	" " " " " " " " " " 1.30 P.M.
"	Adult ♀	"	" " " " " " " " " " 3 P.M.
"	Very large adult ♀	"	" Taken [illegible] crevice in rocks in fairly shallow water at about 3.30 P.M.
"	Adult ♂	"	" Taken among rocks in shallow water at about 4.45 P.M.
30/7/60;	Large adult ♀	"	" " " " " " " " " 12.30 P.M.
"	Large adult ♀	"	" " " " " " " " " 1.15 P.M., after a hard struggle.
"	Half grown ♀	"	" Taken in a hole among rocks in shallow water at about 1.20 P.M.
"	Very large adult ♀	"	" Taken among rocks in shallow water at about 4.30 P.M. after a hard struggle.
"	Adult ♂	"	" " " " " " " " " " 5.15 P.M.
31/7/60;	Adult ♂	"	" Taken in grass on dry land at edge of lake at about 8.30 A.M.
"	Half grown ♀	"	" Taken in shallow water among rocks at about 8.30 A.M., after very long struggle.
1/8/60;	Adult ♀	"	" " " " " " " " " " 8 A.M.

1/9/60; Half grown ♀; MPWAPWA(?) [illegible], N.R. Taken among rocks in shallow water at about 9.15 A.M.
" Very large adult ♀ " " " " " " " " " " 3.45 P.M.
" adult ♂ " " " " " " " " " " 4 P.M.
" adult ♂ " " Taken among rocks in very shallow water after a hard struggle at about 4.45 P.M.
2/9/60; Large adult ♀ " " Taken among rocks in shallow water at about 2 P.M.
" Large adult ♀ " " " " " " " " " " 5 P.M.
3/9/60; Adult ♂ " " Taken when emerging from rocks in fairly deepish water at about 5.10 P.M.

PSEUDOHAJE GOLDII (BOULENG[illegible]

Description: S.R. 15 across neck, [illegible]
broader than deep so that the s[illegible]
or 3rd & 4th upper labials en[illegible]
$4\frac{1}{4}$ times in the total length; al[illegible]

Colour, above, glossy black, [illegible]
sides of head & end of snout [illegible]
with a black edge which become[illegible]
of the body the shields are entir[illegible]

Length up to about 7 feet or [illegible]

Locality. ITURI FOREST, BEL[illegible]

Habitat. Rain Forest.

Habits. Nocturnal. Arbore[illegible]
she had taken refuge after [illegible]

Breeding: Oviparous.

(KAKAMEGA)
Local name:- LIHIRI.

BITIS NASICORNIS (SHAW, 1802). – Nose-horned Viper.

Description: S.R. 31-47; V. 117-140; S.C. (paired) 12-32; head comparatively narrow and covered with small keeled scales, there being 4-6 series of scales between nasal and rostral, and 14-15 scales across head from eye to eye; there are 2 or 3 enlarged horn-like scales over the internasals, usually with very small scales between them; neck relatively slender; body very stout; tail very short (in ♂♂ ·10 to ·15, in ♀♀ ·07 to ·08 of total length).

Colour, above, head bluish green with a dark lance-shaped mark, with its point on the snout, on top; a vertebral series of pale blue nicked oblongs, each surrounded by and bisected longitudinally by a very narrow bright yellow line, extends all the way down back and tail; there are triangular markings enclosing rectangles of bright red and greyish brown on the sides. Underparts mottled. When freshly sloughed it is a very gaudy and beautifully marked snake.

Length usually up to about 3'-6", but longer have been recorded.
– 6/10/59; KAKAMEGA FOREST, K.C.; ♀; length 3'-5¼"; tl. 3¾"; girth 9½"; weight 5¼ lbs.

Locality. ITURI FOREST, BELGIAN CONGO; BWAMBA, UGANDA; KAKAMEGA FOREST, K.C..

Habitat. Rain Forest, favouring damp localities.

Habits. Nocturnal. Usually lies up in dense bush, under logs, or often coiled up in thickets or even trees up to at least 7 feet off the ground. Much more placid than B.A. ARIETANS, though rather less so than B.G. GABONICA, and can usually be picked up with bare hands when on the ground without much protest. Puffs loudly when angry.

Breeding. Ovo-viviparous. Have been juvenile taken at ÉPULU, ITURI FOREST, BELGIAN CONGO on the 18/3/54.–

16/8/54; ♀, EPULU, ITURI FOREST, BELGIAN CONGO
21/8/54; ♂, MAYALA " " " " K[illegible]
22/8/54; ♂ " " " " " [illegible]
30/8/54; ♀ " " " " "
31/8/54; ♀ " " " " "
" ♀ " " " " "
1/9/54; ♂ " " " " "
" ♀ " " " " " F[illegible]
6/9/54; ♂ " " " " " A[illegible]
8/9/54; ♂ " " " " "
11/9/54; ♂ " " " " " Ver[illegible]
10/9/59; ♀, KAKAMEGA FOREST, K.C., Juven[illegible]
12/9/59; ♂ " " " adult
15/9/59; ♀ " " " "
16/9/59; ♀ " " " "
18/9/59; ♀ " " " "
28/9/59; ♀ " " " Large a[illegible]
29/9/59; ♀ " " " Adult.
2/10/59; ♀ " " " Fair si[illegible]
6/10/59; ♂ " " " adult.
" ♀ " " " "
" ♀ " " " Very larg[illegible]
11/10/59; ♀ " " " " "
12/10/59; ♀ " " " Large [illegible]
29/8/61; ♂ " " " Half gr[illegible]
30/8/61; ♂ " " " adult.
2/9/61; ♂ " " " "
5/9/61; ♂ " " " "
7/9/61; ♂ " " " Fair size
8/9/61; ♀ " " " " "
" ♀ " " " " "
9/9/61; ♂ " " " " "
11/9/61; ♀ " " " adult.
15/9/61; ♂ " " " Fair size.

<u>Food</u>.– Mammals.– E.g. 1/9/54; MAYALA, ITURI FOREST, BELGIAN CONGO; large ♂ on being taken in grass in the village disgorged a freshly swallowed House Rat.– Specimens in captivity ate dead rats freely, but refused live amphibians.–

<u>Poison</u>. Probably similar to <u>B.G. GABONICA</u>, i.e. a combination of haemotoxin and neurotoxin.

<u>Total up to 24/10/61</u>.– 62 (27♂♂, 35♀♀).
<u>Total up to 3/10/63</u>.– 77 (31♂♂, 46♀♀).

60; large ♂ on being taken in grass in cunens in Captivity ate dead rats

haemotoxin and neurotoxin.

16/8/54; ♀, EPULU, ITURI FOREST, BELGIAN CONGO. Half grown. Taken in forest in morning.
21/8/54; ♂ MAYALA " " " " Very large adult. Taken under big log in afternoon.
22/8/54; ♂ " " " " " Adult. Taken in grass near a stream in the forest.
30/8/54; ♀ " " " " " " Taken in grass on the edge of the village in the evening.
31/8/54; ♀ " " " " " " " " " " " " " " " " "
" ♀ " " " " " " " " " " " " " " " " "
1/9/54; ♂ " " " " " " " " " " " " " " " " "
" ♀ " " " " " Fair size. " " " " " " " " " " "
6/9/54; ♂ " " " " " Adult. Taken 6 foot up in fork of tree in forest at about 3 P.M.
8/9/54; ♂ " " " " " " Taken on the edge of the forest near the Pygmy village at about 6-30 P.M.
11/9/54; ♂ " " " " " Very large adult. Taken in the forest at about midday.
10/9/59; ♀ KAKAMEGA FOREST, K.C. Juvenile. Taken coiled up about 6 ft off the ground in forest at about 10-30 A.M.
12/9/59; ♂ " " " Adult. Taken on a bush about 4 ft above the ground at about 10 A.M.
15/9/59; ♀ " " " " Taken in thick stuff under a bush at about 1 P.M.
16/9/59; ♀ " " " " Taken under a heap of rubbish in a shamba at about 12:30 P.M.
18/9/59; ♀ " " " " Taken in a bush at about 11-30 A.M.
28/9/59; ♀ " " " Large adult. Taken in a dense heap of rotting rubbish at about 9 A.M.
29/9/59; ♀ " " " Adult. Taken in undergrowth at about 8-45 A.M.
2/10/59; ♀ " " " Fair size. Taken on a bush in a thicket about 5 ft above the ground at about 11-45 A.M.
6/10/59; ♂ " " " Adult. Taken about 7 ft above the ground in a thicket at about 9-15 A.M.
" ♀ " " " " Taken about 6 ft above the ground in a thicket at about 9-45 A.M.
" ♀ " " " Very large adult. Taken in thick reeds at about 1-30 P.M.
11/10/59; ♀ " " " " " " Taken in dense undergrowth at about 9:30 A.M.
12/10/59; ♀ " " " Large adult. Taken in matted grass at about 1 P.M.
29/8/61; ♂ " " " Half grown. Taken about 6 ft up in a bush at about 6 P.M.
30/8/61; ♂ " " " Adult. Taken under a bush at about 1 P.M.
2/9/61; ♂ " " " " Taken in dense longish grass at about 2 P.M.
5/9/61; ♂ " " " " Taken about 7 ft up in a dense thicket at about 5-30 P.M.
7/9/61; ♂ " " " Fair size. Taken about 6 ft up in a sapling in riverine forest at about 1-40 P.M.
8/9/61; ♀ " " " " " Taken about 3 ft off the ground in riverine thicket at about 2 P.M.
" ♀ " " " " " Taken coiled up on a treestump in a patch of forest at about 2-10 P.M.
9/9/61; ♂ " " " " " Taken in a bush by a stream at about 2-50 P.M.
11/9/61; ♀ " " " Adult. Taken in undergrowth in forest at about 1-45 P.M.
15/9/61; ♂ " " " Fair size. Taken about 3' above ground in a riverine thicket at about 9-30 A.M.

7/9/61; ♀; KAKAMEGA FOREST, K.C.: Fair size. Taken in a small dense bush at about 3·30 P.M.
15/9/61; ♂ " " " Fair size. Taken about 4' up in thick hedge at about 12·40 P.M.
20/9/61; ♀ " " " Half grown. Taken in a thicket at about 10·45 A.M.
" ♀ " " " Very large adult. Taken under a log in a thicket at about 1·10 P.M.
21/9/61; ♀ " " " Very large adult. Taken on the ground in a thicket at about 10·15 A.M.
22/9/61; ♂ " " " Fair size. Taken about 5' up in riverine thicket at about 11·30 P.M.
" ♂ " " " Fair size. Taken on the ground under brushwood at about 12·15 P.M.
28/9/61; ♀ " " " Adult. Taken coiled up in foot-high grass at about 3 P.M.
29/9/61; ♂ " " " Adult. Taken on the ground in a secondary thicket at about 10·30 A.M.
" ♀ " " " Adult. Taken among "CANNA" lilies in a flower bed at about 4·25 P.M.
30/9/61; ♀ " " " Fair size. Taken in shortish grass in a valley at about 11·30 A.M.
2/10/61; ♀ " " " Very large adult. Taken in dense grass at about 3·10 P.M.
3/10/61; ♀ " " " Half grown. Taken about 4 ft up in a thicket at about 2 P.M.
4/10/61; ♀ " " " Juvenile. Taken in short grass by the side of the motor road at about 6·40 P.M.
5/10/61; ♀ " " " Large adult. Taken about 4 ft up in a thicket at about midday.
9/10/61; ♂ " " " Adult. Taken under a log in a dense riverine thicket at about 2·10 P.M.
11/10/61; 1♂ " " " Juvenile. Taken in a clump of one foot high grass at about 9·50 A.M.
13/10/61; 1♂ " " " Fair size. Taken in a small bush at about 10 A.M.
16/10/61; ♂ " " " Adult. Taken in a very dense thicket at about 9 A.M.
" ♂ " " " Half grown. Taken about 7 feet up in a thicket at about 9·45 A.M.
" ♀ " " " Fair size. Taken about 5 feet up in a thicket at about 2·40 P.M.
" ♂ " " " Half grown. Taken on the ground in a thicket at about 3·30 P.M.
" ♂ " " " Adult. Taken about 6 feet up in a thicket at about 4·10 P.M.
" ♀ " " " Large Adult. Taken in small thick bush at about 4·30 P.M.
20/10/61; ♀ " " " Juvenile. Taken in a patch of 1 foot high grass at about 1·40 P.M.
" ♀ " " " Adult. Taken in short grass near a thicket at about 2 P.M.
" ♂ " " " Half grown. Taken in a small bush at about 3 P.M.
24/10/61; ♀ " " " Very large adult. Taken under dead grass in a valley at about 12·50 P.M.
28/9/63; ♀ " " " Adult. Taken in dense undergrowth at about 9 A.M.
" ♂ " " " Adult. Taken in a bush at about 9·10 A.M.
29/9/63; ♂ " " " Adult. Taken about 7' up in undergrowth at about 9·50 A.M.
" ♀ " " " Adult. Taken ten feet up in a tree among creepers at about 11·60 A.M.
30/9/63; ♀ " " " Half grown. Taken about 6' up in a bush at about 12·45 P.M.
2/10/63; ♀ " " " Adult. Taken about 9' up in a thicket at about 2 P.M.

(Continued on Page 128.)

(KAKA[illegible]

Local name. — NAMA[illegible]

ATHERIS SQUAMIGER (HAL[illegible]

Description: Head broad, fla[illegible] keeled scales; body modera[illegible] keeled; body laterally rathe[illegible] eye with vertically elliptic p[illegible] heavy, anal entire.

Colour, above, dull green with[illegible] olive green.

Length up to over 2 feet; l[illegible]
— ♀; KAKAMEGA FOREST; 28/9/[illegible]
♀; KAKAMEGA FOREST; 28/9/61; length 2[illegible] 3 1/16", weight 8 oz.—

Locality. KAKAMEGA FOREST, [illegible]

Habitat. Rain Forest and it[illegible]

Habits. Usually found on[illegible] ground, or occasionally l[illegible] relies on immobility to con[illegible] considering its size. Sho[illegible]

[illegible]

Breeding. ovo-viviparous.

t 9.55 A.M.
t about 10.05 A.M.

BITIS NASICORNIS (SHAW, 1802). — Nose-horned Viper.

(Continued from Page 97.)

8/10/63; ♀;	KAKAMEGA FOREST, K.C.			Juvenile, recently born. Taken in a tuft of shortish grass at about 2.30 P.M.
10/10/63; ♀	"	"	"	Large adult. Taken in a dense bush at about 1.50 P.M.
" ♀	"	"	"	Adult. " " " " " " " 3.05 P.M.
12/10/63; ♂	"	"	"	Juvenile. " " " " " " " 10.45 A.M.
13/10/63; ♀	"	"	"	Half grown. Taken in undergrowth about 1'-6" up at about 10.10 A.M.
14/10/63; ♀	"	"	"	Adult. Taken in a dense bush at about 5.45 P.M.
18/10/63; ♀	"	"	"	Large adult. Taken in undergrowth at about 5.20 P.M.
22/10/63; ♂	"	"	"	Fair size. Taken about 6' up in a thicket at about 2.10 P.M.
30/10/63; ♀	"	"	"	Quarter grown. Taken in riverine thicket 2 ft up at about 12.30 P.M.

10/9/59; ♂; KAKAMEGA FOREST, KENYA. adult. Taken on sapling in thicket at about 2 P.M.
15/9/59; ♂ " " " Large adult. Taken in a tree in a thicket at about 10.30 A.M.
19/9/59; ♂ " " " Juvenile. Taken in a tree at about 11.45 A.M.
21/9/59; ♂ " " " young adult. Taken in a thicket at about 10.45 A.M.
23/9/59; ♀ " " " Very large adult. Taken in a tree in very thick growth at about 3 P.M.
24/9/59; ♂ " " " Large adult. Taken in a very dense thicket at about 11.30 A.M.
25/9/59; ♂ " " " Adult. Taken in a dense thicket at about 10.45 A.M.
" ♂ " " " Large adult. Taken in a thicket after descending in pursuit of another male at about 11.30 A.M.
" ♀ " " " Very large adult. Taken in a lowish tree at about 2.45 P.M.
28/9/59; ♀ " " " adult. Taken in a thicket at about 11.45 A.M.
" ♀ " " " adult. Taken in a tree at about 4 P.M.
1/10/59; ♂ " " " adult. Taken high up in a Cypress tree at about 11.30 A.M.
3/10/59; ♀ " " " Very large adult. Taken in a dense thicket at about 11.45 A.M.
" ♂ " " " Very large adult. " " " " " " " 12.30 P.M.
6/10/59; ♀ " " " adult. " " " " " " " 11 A.M.
7/10/59; ♀ " " " Very large adult. " " " " " " " 3.30 P.M.
9/10/59; ♀ " " " Large adult. " " " " " " " 11.30 A.M.
10/10/59; ♂ " " " adult. Taken in a tree at about 10.15 A.M.
" ♀ " " " adult. } Taken in the same tree at 11.15 A.M. and 11.30 A.M.
" ♂ " " " adult. } respectively.
2/9/61; ♂ " " " adult. Taken in a tree in dense riverine thicket at about 9.45 A.M.
" ♀ " " " adult. " " " " " " " " " " 10 A.M.
" ♂ " " " adult. " " " " " " " " " " 11.50 A.M.
4/9/61; ♂ " " " Juvenile. Taken in a high dense bush at about 1 P.M.
5/9/61; ♀ " " " adult. Taken high up in a tree at about midday.
" ♀ " " " Half grown. Taken on a bush at about 2 P.M.
6/9/61; ♀ " " " adult. Taken in dense riverine thicket at about 2 P.M. Trouble with "SIAFU."
9/9/61; ♀ " " " adult. " " " " " " " 12.10 P.M.
13/9/61; ♂ " " " adult. } Taken in the same high tree at about 5.15 P.M. and 5.45 P.M.
" ♀ " " " adult. } respectively.
14/9/61; ♂ " " " adult. Taken low down in riverine thicket at about 2.30 P.M.
20/9/61; ♀ " " " Juvenile. Taken in a Cypress tree at about 9.50 A.M.
21/9/61; ♀ " " " adult. Taken in moderate sized tree in riverine thicket at about 9.15 A.M.
22/9/61; ♂ " " " adult. Taken on ground after he had fallen out of Cypress tree at about 1.30 P.M.

24/9/61; ♂; KAKAMEGA FOREST, KENYA. adult. Taken in a high tree at about 2.40 P.M.
25/9/61; ♀ " " " adult. Taken very low down in a thicket at about 10.40 A.M.
26/9/61; ♀ " " " adult. Taken in a cypress tree at about 12.10 P.M.
" ♂ " " " adult. " " " " " " " 12.30 P.M.
" ♀ " " " adult. Taken in a dense thicket at about 12.45 P.M.
27/9/61; ♂ " " " Large adult. " " " " " " " 4.30 P.M.
28/9/61; ♂ " " " adult. Taken in a Cypress tree at about 9.30 A.M.
" ♀ " " " adult. " " " " " " " 2 P.M.
30/9/61; ♂ " " " adult. Taken in a low tree in a riverine thicket at about 9.50 A.M.
" ♀ " " " Large adult. Taken as she was coming down from a high tree at about 10.50 A.M.
2/10/61; ♂ " " " Large adult. Taken in a high tree at about 2.45 P.M.
6/10/61; ♂ " " " adult. } Taken together on a low prickly bush at the edge of a
" ♀ " " " adult. } secondary thicket at about 9.30 A.M.
7/10/61; ♂ " " " adult. Taken on the ground among the branches of a fallen tree at about 9.45 A.M.
16/10/61; ♂ " " " adult. Taken in a very dense thicket at about 9.45 A.M.
17/10/61; ♀ " " " Very large adult. } Taken in the same high tree in a riverine thicket at about
" ♀ " " " Fair size. } 2.50 P.M. and 3.20 P.M. respectively.
20/10/61; ♀ " " " Large adult. Taken fairly high up in a riverine thicket at about 11.30 A.M.
21/10/61; ♂ " " " Large adult. Taken in a tree in a riverine thicket at about 11 A.M.
24/10/61; ♀ " " " adult. Taken in a tree in a riverine thicket at about 1.20 P.M.
28/9/63; ♀ " " " Fair size. Taken in dense forest at about 8.30 A.M.
" ♂ " " " Very large adult. Taken in very dense undergrowth of riverine forest at about 12.35 P.M.
29/9/63; ♂ " " " Fair size. Taken in a riverine thicket at about 1.30 P.M.
" ♂ " " " adult. " " " " " " " 1.35 P.M.
3/10/63; ♀ " " " Juvenile. Taken in a low tree at about 1.30 P.M.
" ♂ " " " Fair size. Taken in a thicket at about 1.45 P.M.
4/10/63; ♂ " " " adult. Taken on the ground inside a store house at about 5.30 P.M.
6/10/63; ♂ " " " adult. Taken in a lowish tree at about 12.30 P.M.
8/10/63; ♀ " " " adult. Taken low down in a large tree overhanging a river at about 11.15 A.M.
" ♂ " " " adult. " " " " " " " " " " " 11.20 A.M.
14/10/63; ♂ " " " adult. Taken in a low tree by a stream at about 2.20 P.M.
16/10/63; ♂ " " " adult. Taken on ground in undergrowth of small isolated bush at about 11.30 A.M.
17/10/63; ♀ " " " adult. Taken in a high bush at about 5 P.M.
19/10/63; ♀ " " " adult. Taken in a tree at about 9.45 A.M.
